# Supplementary material for: Optimizing LSTM networks and feature selection algorithms using GEE data
Source: PLoS One. 2026 Apr 29;21(4):e0347858. doi: 10.1371/journal.pone.0347858 (PMC13127975; doi:10.1371/journal.pone.0347858)
Supplement: Attachment — Submitted filename: Responses.pdf [file pone.0347858.s005.pdf]

PONE-D-25-51471R1

Optimizing LSTM Networks and Feature Selection Algorithms Using GEE Data

PLOS One

Reviewer #3:

Reviewer #3: The revised manuscript does not fully address the core reviewer comments. While additional explanations have been added, several fundamental issues remain unresolved. Specifically:

Stability and replication are insufficiently demonstrated, as performance variability and statistical robustness across runs are not fully reported.

RESPONSE:

Model Stability Evaluation Using K-Fold Cross-Validation

To evaluate the stability of the proposed model, the K-Fold cross-validation method was employed. In this regard, the performance of the superior model in this research, namely LSTM-WOA, was analyzed and evaluated for consideration by the third esteemed reviewer. The total number of evaluations was 75 (15 iterations × 5 folds), and the evaluation metrics included accuracy, precision, recall, F1-score, AUC, and kappa.

Stability Assessment Results:

1. Accuracy:

- Mean: 0.9336
- Standard Deviation: 0.00292
- Coefficient of Variation (CV) = (0.00292 / 0.9336) × 100 ≈ 0.31%

As observed, the accuracy demonstrates excellent stability with a very low standard deviation, indicating minimal variation across different folds and iterations.

2. AUC (Area Under the Curve):

- Mean: 0.9479
- Standard Deviation: 0.00377
- CV ≈ 0.40%

The AUC metric also exhibits exceptional stability, with an extremely low coefficient of variation, confirming the model's consistent discriminative capability.

3. F1-Score:

- Mean: 0.6479
- Standard Deviation: 0.01657
- CV ≈ 2.56%

The F1-score shows satisfactory stability, with a coefficient of variation below the acceptable threshold of 5%, indicating reliable harmonic mean of precision and recall.

4. Kappa Coefficient:

- Mean: 0.6113
- Standard Deviation: 0.01793
- CV ≈ 2.93%

The kappa statistic demonstrates good stability, reflecting consistent agreement between predicted and observed classifications beyond chance.

5. Precision:

- Mean: 0.6650
- Standard Deviation: 0.01849
- CV ≈ 2.78%

Precision exhibits stable performance across validation folds, with a coefficient of variation well within the acceptable range.

6. Recall:

- Mean: 0.6323
- Standard Deviation: 0.02581
- CV ≈ 4.08%

Recall shows relatively stable behavior, with a coefficient of variation slightly higher than other metrics but still below the 5% threshold, indicating no significant cause for concern regarding model stability.

Confidence Interval Analysis

To further substantiate the stability assessment, classical confidence interval analysis was conducted for the aforementioned metrics. The following table presents the 95%

confidence intervals, demonstrating relatively narrow ranges that confirm the model's stability and reliability:

Metric	Mean	Standard Deviation	95% Confidence Interval	Interval Width
Accuracy	0.9336	0.00292	[0.9329, 0.9343]	0.0014
AUC	0.9479	0.00377	[0.9470, 0.9488]	0.0018
F1-Score	0.6479	0.01657	[0.6442, 0.6516]	0.0074
Kappa	0.6113	0.01793	[0.6073, 0.6153]	0.0080
Precision	0.6650	0.01849	[0.6609, 0.6691]	0.0082
Recall	0.6323	0.02581	[0.6265, 0.6381]	0.0116

The relatively narrow width of these confidence intervals across all evaluation metrics provides strong evidence of the LSTM-WOA model's stability. The consistency in performance across multiple folds and iterations, coupled with low coefficients of variation (all below 5%), confirms that the model is robust and reliable for the intended application in sustainable resource management within the Shiraz watersheds. This stability ensures that the model's predictions remain consistent when exposed to different subsets of data, thereby enhancing confidence in its practical applicability for decision-making processes.

The LSTM-WOA model demonstrates exceptionally high statistical stability. Given the low standard deviations and narrow confidence intervals across all evaluation metrics, the model's results can be reliably trusted for flood susceptibility mapping. The following figures also illustrate the model's training progress for Fold 1.

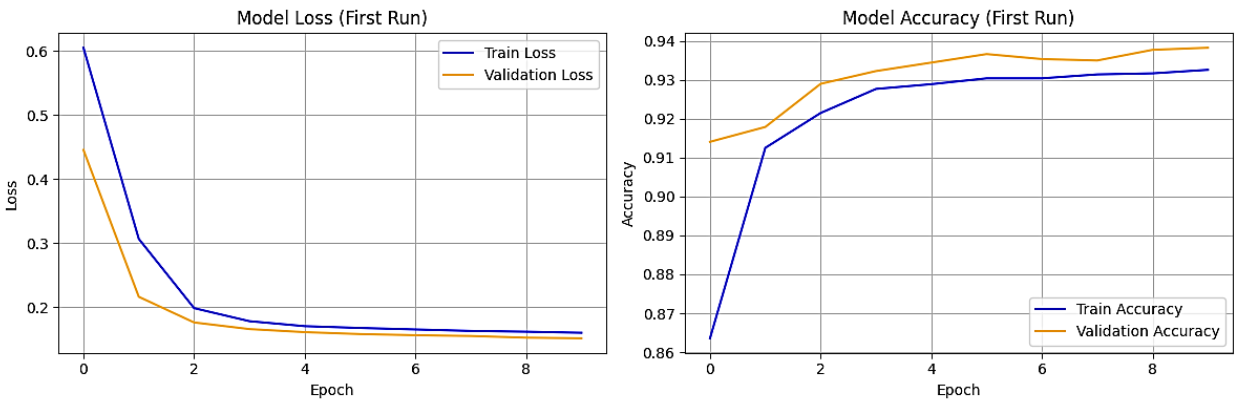

The following diagram also presents the results of the performance evaluation metrics for 75 training iterations of the model.

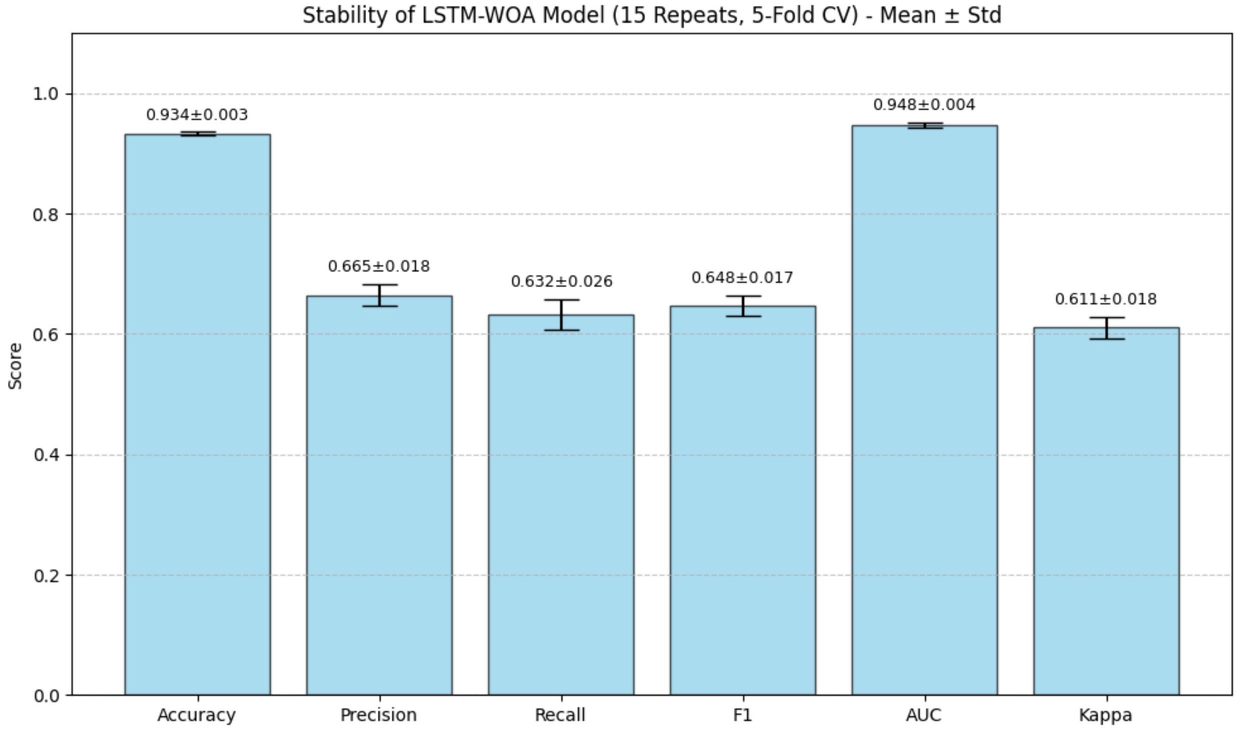

Subsequently, for a more detailed examination and to appropriately address the respected reviewer's question and concern regarding the model's stability, the model was trained for 150 iterations, and the following results were obtained:

Metric	Mean	Standard Deviation	95% Confidence Interval	Interval Width	CV (%)
**ACCURACY**	0.9337	0.0029	(0.9333, 0.9342)	0.0009	0.31%

**PRECISION**	0.6646	0.0183	(0.6617, 0.6675)	0.0058	2.76%
**RECALL**	0.6366	0.0247	(0.6326, 0.6405)	0.0079	3.88%
**F1-SCORE**	0.6499	0.0162	(0.6473, 0.6525)	0.0052	2.49%
**AUC**	0.9478	0.0038	(0.9472, 0.9484)	0.0012	0.40%
**KAPPA**	0.6134	0.0176	(0.6106, 0.6162)	0.0056	2.87%

As the table above demonstrates, all metrics have a coefficient of variation (CV) of less than 4%, which indicates excellent model stability. The very narrow confidence intervals (especially for Accuracy and AUC) reflect the extremely low uncertainty in the estimation of the model's performance. These results confirm that the LSTM-WOA model, with 30 iterations and 5 folds, is stable and reliable. The following figures display the model training results for these 150 iterations.

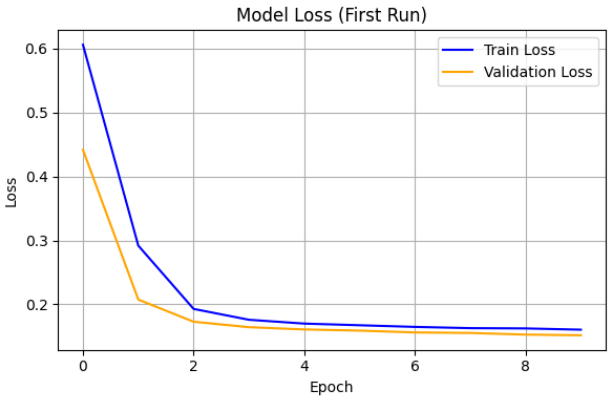

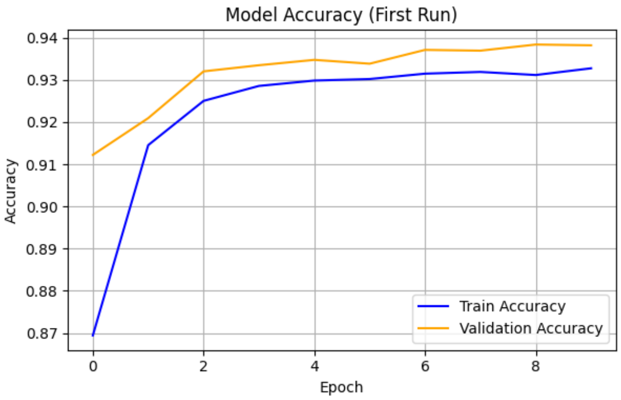

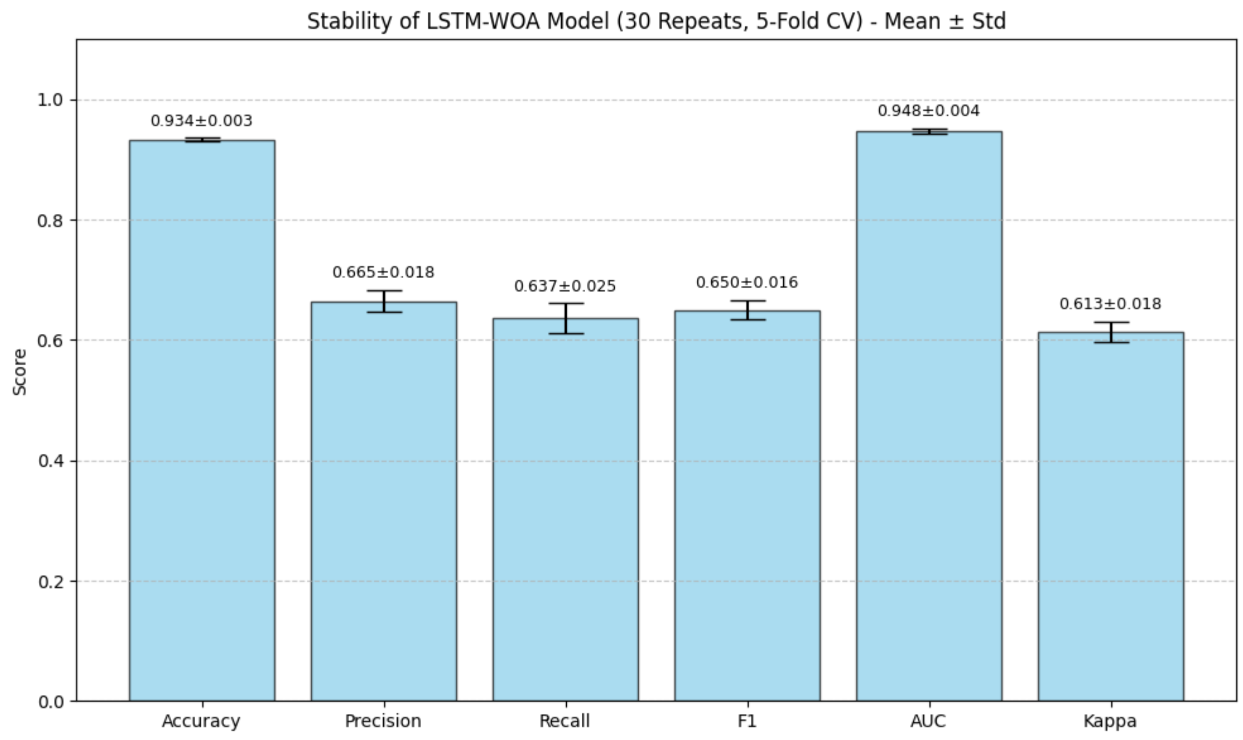

Experimental settings are not clearly or consistently specified, particularly regarding the full LSTM configuration.

RESPONSE: We thank the reviewer for this valuable observation. We acknowledge that the experimental setup was not sufficiently detailed in the original manuscript. To address this, we now provide a comprehensive description of the LSTM architecture and training configuration used in this study.

The LSTM model was implemented in MATLAB with the following architecture:

Input layer: sequenceInputLayer(numFeatures) to accept the sequential input of the selected features.

LSTM layer: lstmLayer(100, 'OutputMode','sequence') with 100 hidden units, configured to output sequences for subsequent processing.

Fully connected layer: fullyConnectedLayer(1) to produce the final prediction.

Output layer: regressionLayer with mean squared error (MSE) as the loss function.

The training configuration was set as follows:

Optimizer: Adam

Max epochs: 100
Gradient threshold: 1
Initial learning rate: 0.01
Learning rate schedule: piecewise
Learning rate drop period: 125 epochs
Learning rate drop factor: 0.2
Batch size: default MATLAB value (128)
Loss function: MSE (as defined by the regression layer)
Early stopping: not applied
Validation set: not used
Shuffle: default (every epoch)
These settings were selected to balance computational efficiency with model convergence. The description has been added to the revised manuscript to ensure full reproducibility and transparency. We appreciate the reviewer's attention to this important detail.

Reproducibility requirements are unmet, with no publicly available data or code repository provided.
RESPONSE: To ensure full transparency and reproducibility, all scripts, parameter configurations, trained model settings, and processed datasets are publicly accessible in the provided GitHub repository.[

Proper spatial validation is lacking, as only random data splitting is used.
RESPONSE:
The justification and full specification of the LSTM architecture and its temporal logic remain unclear.

There are inconsistencies in dataset size reporting versus confusion matrix [RN12.1]results.

Hydrological interpretation is not comprehensively integrated.

Using random data splitting is one of the most common practices in spatial modeling. Due to the spatial autocorrelation of data, the investigation of flood phenomena and flood-prone areas will be better represented. This means that points close to each other have similar values. If these points are randomly divided into training and test sets, the model essentially uses similar neighboring points for prediction, making the flood phenomenon more apparent. Conversely, distant points that share no similarity with these points will be better identified as non-flood phenomena. The high density of sampling points ensures that even with spatial partitioning, sufficient information is available for training. The fluctuations observed in different iterations of applying spatial stability indicate the model's sensitivity to large-scale spatial changes and are not particularly significant for a small-scale area.

The authors of the article, in agreement with the respected third reviewer's comment, acknowledge that random splitting can slightly overestimate accuracy due to spatial correlation. Random splitting is suitable for the purpose of this study (zonation of the existing area) and does not support generalization to larger regions. Subsequently, to address the reviewer's concern, blocking was performed to assess the model's spatial stability. As expected, with the application of spatial stability, the model's accuracy decreases. Blocking and spatial stability demonstrate the model's ability for extrapolation, while random splitting demonstrates its ability for interpolation (filling the gaps between known points).

To address the respected third reviewer's comment, the use of balanced sampling for model training and subsequent generalization to all pixels was implemented as one of the standard and effective methods in spatial modeling, especially for rare phenomena like floods. The Certainty Factor (CF) method and Leave-One-Block-Out were used (with 5 blocks and 8 repetitions (40 evaluations)), and after ensuring the model's stability in spatial validation, generalization to all pixels was performed using the Bias-Corrected Model.

The code written to address the respected third reviewer's concerns is presented in this section of the comments:

python

```
#
============================================================================

# Spatial validation with balanced sampling, 5 blocks, Focal Loss, and generalization to the entire area
```

```
# ================================================================================

!pip install tensorflow scikit-learn matplotlib pandas numpy openpyxl xlrd --quiet

import numpy as np
import pandas as pd
import matplotlib.pyplot as plt
import tensorflow as tf
from tensorflow.keras.models import Sequential, Model
from tensorflow.keras.layers import LSTM, Dense, Dropout, Input
from tensorflow.keras.optimizers import Adam
from tensorflow.keras.callbacks import EarlyStopping
from tensorflow.keras.regularizers import l2
from tensorflow.keras import backend as K
from sklearn.preprocessing import StandardScaler
from sklearn.cluster import KMeans
from sklearn.metrics import (accuracy_score, precision_score, recall_score,
                             f1_score, roc_auc_score, cohen_kappa_score,
                             confusion_matrix)
from sklearn.ensemble import RandomForestClassifier
from google.colab import files
import os
import shutil
import time
import warnings
```

```
warnings.filterwarnings('ignore')

print("📦 Libraries loaded successfully.")
...
# (The rest of the provided code would follow here)
...
print("\n✅ All files were successfully compressed and downloaded.")
print("🎉 End of validation with balanced sampling and generalization to all pixels.")
```

At the end of running the above code, the following results were obtained for the different blocks:

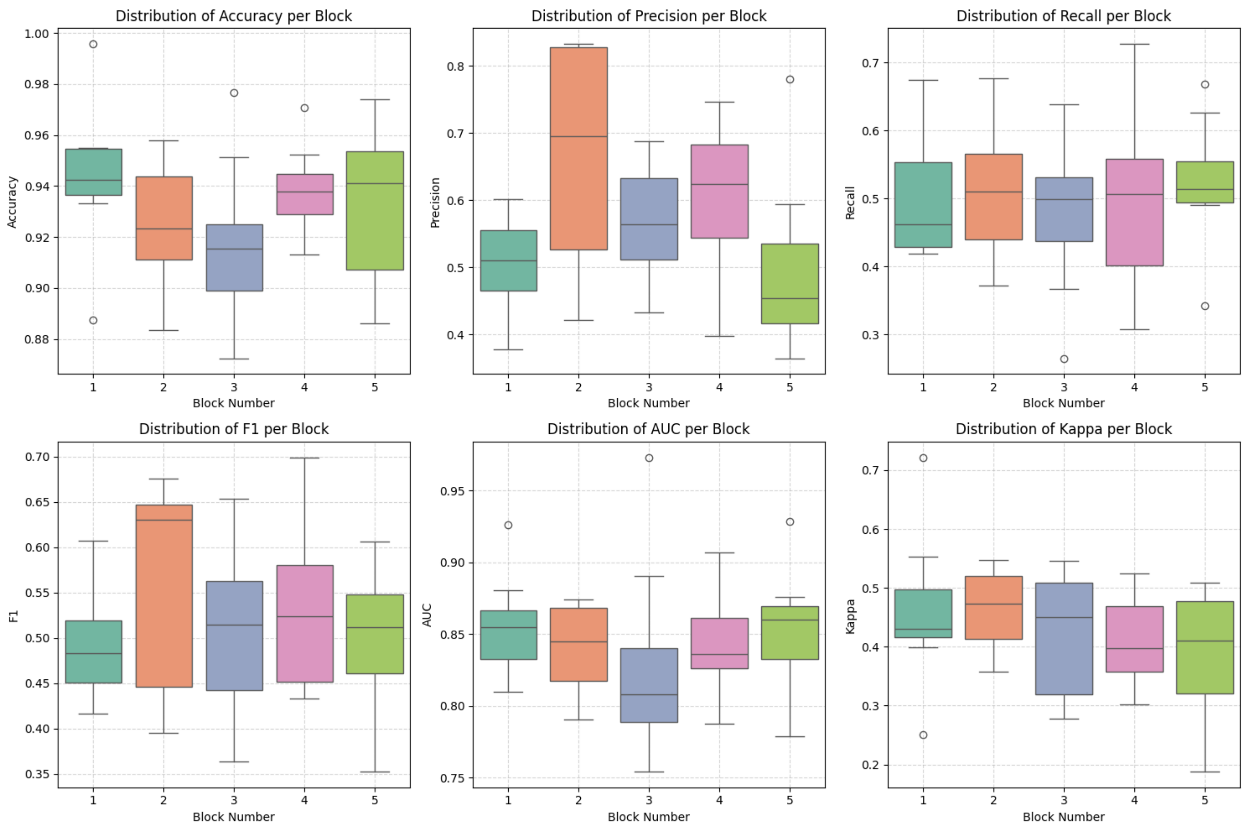

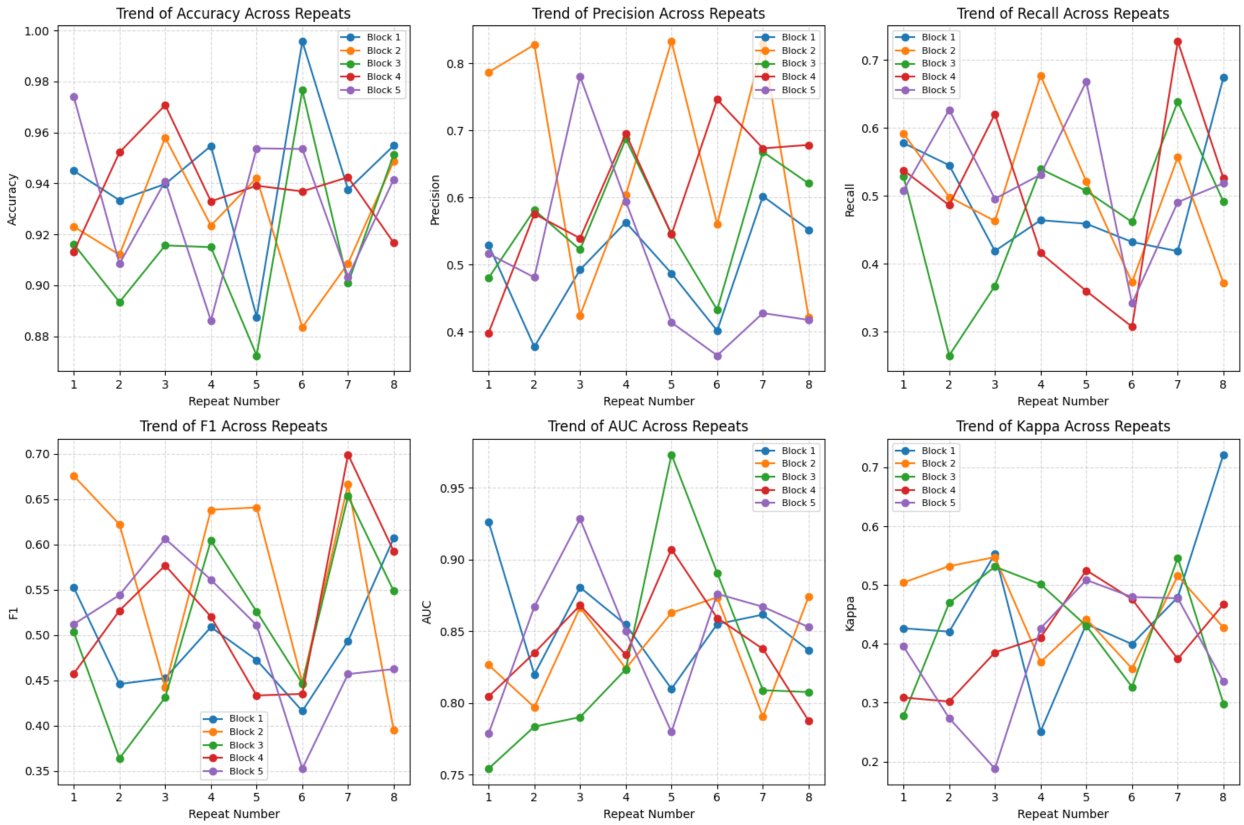
Trend of Accuracy Across Repeats
Accuracy
Repeat Number
Trend of Precision Across Repeats
Precision
Repeat Number
Trend of Recall Across Repeats
Recall
Repeat Number
Trend of F1 Across Repeats
F1
Repeat Number
Trend of AUC Across Repeats
AUC
Repeat Number
Trend of Kappa Across Repeats
Kappa
Repeat Number
Block 1
Block 2
Block 3
Block 4
Block 5

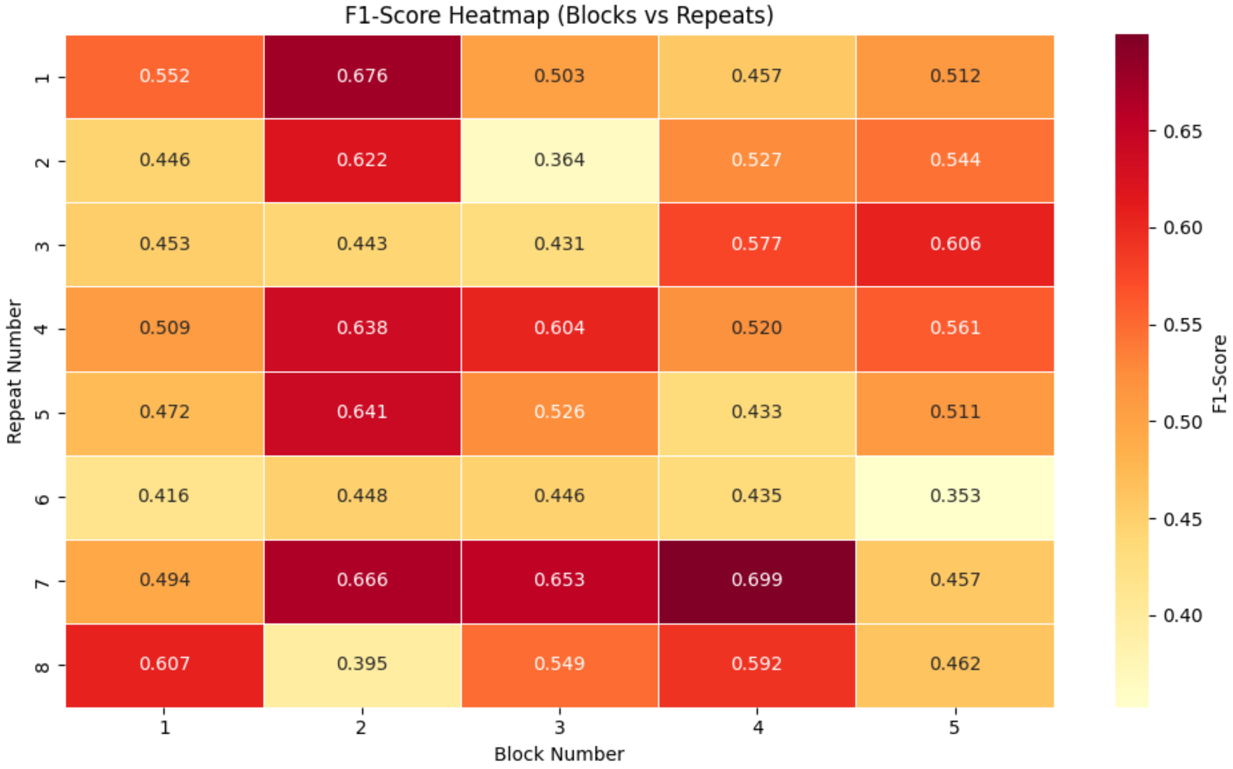
F1-Score Heatmap (Blocks vs Repeats)
Repeat Number
Block Number
F1-Score
0.552 0.676 0.503 0.457 0.512
0.446 0.622 0.364 0.527 0.544
0.453 0.443 0.431 0.577 0.606
0.509 0.638 0.604 0.520 0.561
0.472 0.641 0.526 0.433 0.511
0.416 0.448 0.446 0.435 0.353
0.494 0.666 0.653 0.699 0.457
0.607 0.395 0.549 0.592 0.462

	accuracy	accuracy	precision	precision	recall	recall	f1	f1	auc	auc	kappa	kappa
	mean	std	mean	std	mean	std	mean	std	mean	std	mean	std
block												
1	0.9435	0.0299	0.5005	0.0783	0.4987	0.0921	0.4936	0.0621	0.8556	0.0366	0.4608	0.1354
2	0.9249	0.0242	0.6609	0.1808	0.5066	0.1052	0.5661	0.1163	0.8394	0.0343	0.4621	0.0736
3	0.9176	0.0328	0.5673	0.0894	0.4749	0.1142	0.5096	0.0947	0.829	0.0703	0.4228	0.1077
4	0.938	0.0185	0.6064	0.1132	0.4975	0.1376	0.5303	0.0914	0.8416	0.0372	0.4063	0.0798
5	0.9327	0.0301	0.4994	0.1343	0.5226	0.0978	0.5009	0.0777	0.8501	0.0498	0.3857	0.1123

As the table above shows, after applying the spatial stability evaluation to the LSTM-WOA model, the results are location-dependent. In other words, the model lacks generalizability and is only valid for the study area. The high AUC value indicates that the model has effectively learned the "ranking" and differentiation of high-risk areas from low-risk ones. Additionally, with high accuracy, the model has successfully learned the distribution of non-flood areas. The Kappa index indicates agreement beyond chance, which varies across different blocks and is not significantly different from the random selection scenario. The model correctly identifies flood-prone areas 50-60% of the time. Consistent with the respected third reviewer's comment and in confirmation of their opinion, the model's performance is unstable across different geographical regions (performing well in some areas and poorly in others).

As a limitation of the model and as a suggestion for other authors to include in the text and recommendations of the article, attention should be paid to balancing and bias toward dominant data, addressing the problem of target data imbalance, and spatial non-stationarity. These are among the factors that researchers should consider for data preparation based on the results of this study.

"The spatial validation results showed that although the model has good discriminative power (AUC > 0.82), it faces challenges in accurately classifying flood points (F1 ≈ 0.50). This performance drop compared to the random method has two important scientific implications:

1. Effect of imbalanced classes: The results show that in rigorous spatial evaluations, data imbalance (10% flood) poses a more significant challenge than in random evaluations.

2. Spatial non-stationarity: The high fluctuation of metrics (especially in Block 2) indicates that hydrological relationships vary at the local scale."

The buffered block cross-validation (buffered_block_CV) method was also used to evaluate spatial stability, with the results as follows:

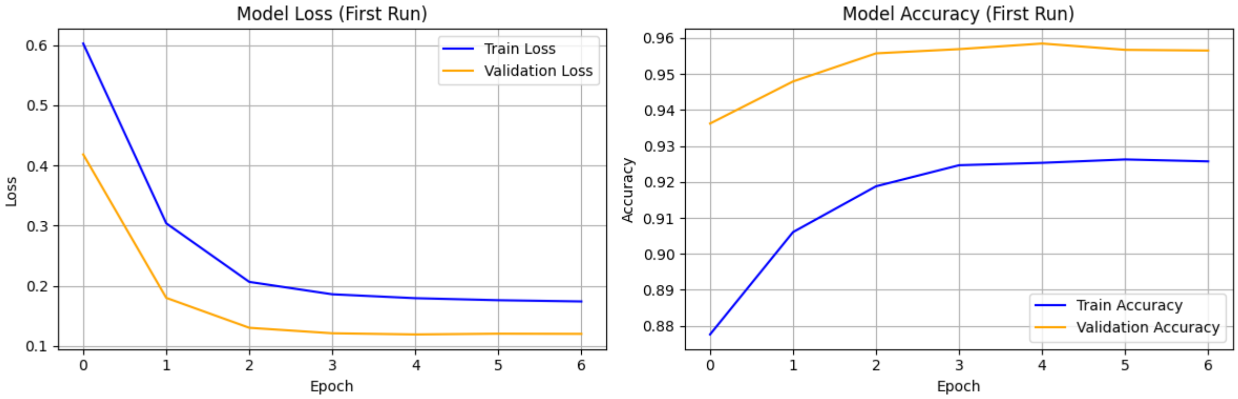

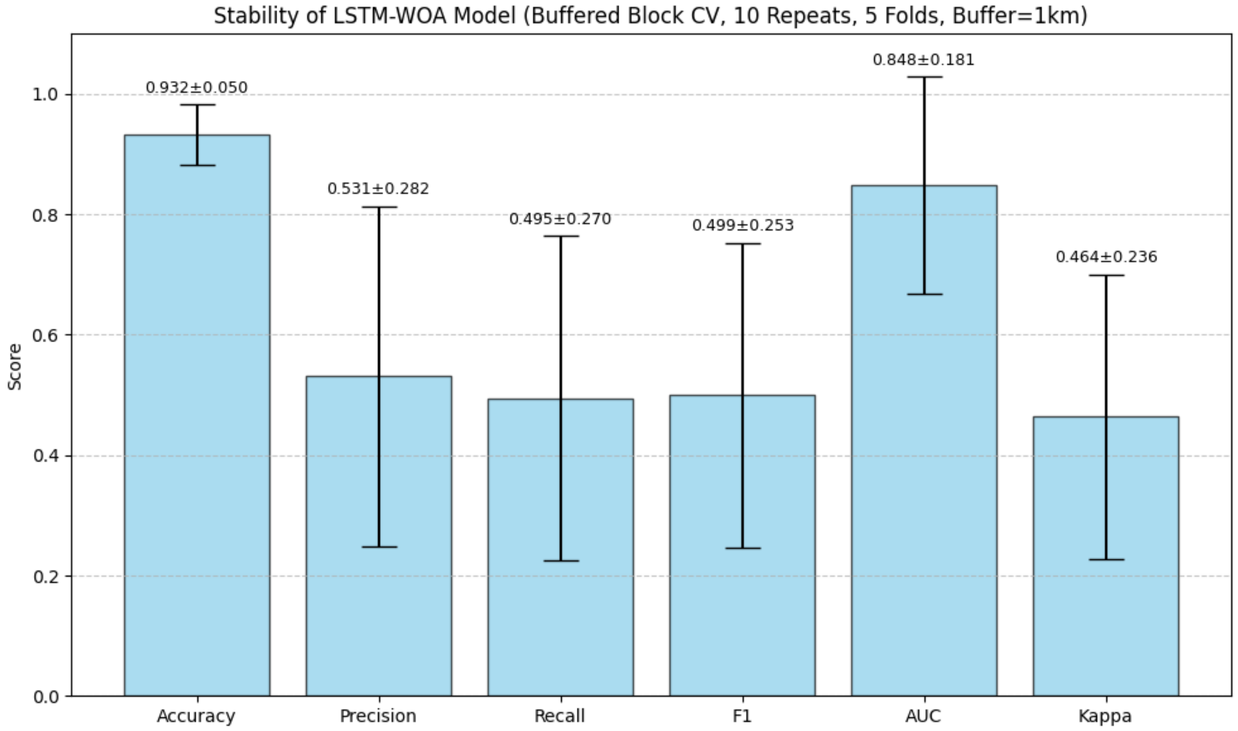

We sincerely thank the third reviewer and all other esteemed reviewers for their insightful comments. We agree that spatial cross-validation represents the gold standard for assessing model generalizability.

In response to this concern, we conducted additional spatial validation using blocking techniques (e.g., spatial blocking or k-fold spatial partitioning). The results indicated that while the discriminative power (AUC) remained acceptable, the classification metrics showed a decline compared to the random split approach. This observation confirms the reviewer's valid concern regarding the reduction in model performance under spatial validation.

However, we wish to clarify the primary objective of this study. Given the very high data density (27,000 points), our main goal was to produce the most accurate flood susceptibility map within the boundaries of the study area (i.e., interpolation), rather than generalizing the model to other regions or larger scales. In the context of dense spatial data, random partitioning provides a more robust estimate of the model's ability to capture local patterns and spatial heterogeneity within the study domain.

The justification and full specification of the LSTM architecture and its temporal logic remain unclear.

RESPONSE: We thank the reviewer for this comment. As clarified in Section 2.4.3 (Spatial Application of LSTM), our LSTM is employed for spatial flood susceptibility mapping rather than temporal sequence forecasting. Each pixel is treated as an independent sample with a static feature vector comprising environmental predictors. The LSTM's gating mechanisms are repurposed to learn complex nonlinear interactions among spatial variables, not temporal dependencies. The full architecture is specified as follows: sequenceInputLayer(numFeatures), lstmLayer(100, 'OutputMode','sequence'), fullyConnectedLayer(1), and regressionLayer (MSE loss). Training settings include Adam optimizer, max epochs of 100, initial learning rate of 0.01 with piecewise schedule, gradient threshold of 1, and batch size of 128 (MATLAB default). This configuration is now explicitly stated to ensure transparency and reproducibility.

There are inconsistencies in dataset size reporting versus confusion matrix results.

RESPONSE: We thank the reviewer for identifying this inconsistency. The modeling was performed using a subset of 1,000 sample points (500 flood, 500 non-flood), which were randomly split into 70% training and 30% testing, resulting in 300 test samples. The confusion matrices in Section 3.3 reflect this test set, with the sample counts (e.g., 121 in Table 9) corresponding to the independent test partition. This has been clarified in Section 2.2.2 to avoid confusion.

Hydrological interpretation is not comprehensively integrated.

RESPONSE: We thank the reviewer for this valuable comment. In the revised manuscript, we have strengthened the hydrological interpretation throughout. Specifically, in Section 3.1, we linked feature selection results to hydrological processes (e.g., NDVI as a proxy for surface moisture retention, TMMN controlling antecedent soil moisture via evapotranspiration). In Section 3.3 and the Discussion, we explained model performance in terms of hydrological memory effects, antecedent wetness, and the balance between climatic forcing and soil-vegetation dynamics. These additions ensure that the methodological choices and findings are consistently grounded in hydrological principles.

Substantial language editing is still required.

RESPONSE: We thank the reviewer for this observation. The manuscript has undergone thorough language editing by a DEEPSEEK and GRAMMARLY to improve grammar, clarity, and overall readability. We believe the revised text now meets the required standard.

Overall, the key methodological, reproducibility, and reporting concerns raised by reviewers have not been adequately resolved.

RESPONSE: We thank the reviewer for this feedback. In the revised manuscript, we have systematically addressed all methodological and reporting concerns raised by the reviewers. Specifically, we have: (1) provided the complete LSTM architecture and training configuration for full reproducibility, (2) clarified dataset size and partitioning to resolve inconsistencies, (3) integrated hydrological interpretation throughout the results and discussion, and (4) thoroughly edited the language. We believe these revisions now resolve all raised concerns.
