# Supplementary material for: Optimizing LSTM networks and feature selection algorithms using GEE data
Source: PLoS One. 2026 Apr 29;21(4):e0347858. doi: 10.1371/journal.pone.0347858 (PMC13127975; doi:10.1371/journal.pone.0347858)
Supplement: Attachment — Submitted filename: Responses.docx [file pone.0347858.s003.docx]

Dear Editor,

Thank you for your valuable comments regarding the potential copyright issues in the figures. We appreciate your diligence in ensuring compliance with the journal's CC BY 4.0 license policy.

We have carefully reviewed the figures in question and would like to clarify their origins:

**For Figures 1 & 15 (Maps and Satellite Images):**

We confirm that all map and satellite imagery presented in these figures were generated by the authors using **exclusively free and openly accessible data sources** that are compatible with open publication. The base data (including satellite imagery and elevation data) were sourced from publicly available repositories such as the Copernicus program (for Sentinel data) and processed within the Google Earth Engine and QGIS platforms. No proprietary or copyrighted map tiles (e.g., from Google Maps, Street View, or Earth) were used. We have revised the caption for **Figure 15** to explicitly state this.

**For Figures 2 & 5 (and all other analytical figures):**

We confirm that these figures are **original creations by the authors**. They represent direct outputs of our research analysis, including schematic diagrams, feature importance plots, result curves, and confusion matrices, generated programmatically from our code and data. They are not reproduced from any copyrighted source.

**Summary of Revisions Made:**

- **Caption for Fig. 1:** We have ensured it clearly indicates the use of free and openly accessible data sources.
- **Caption for Fig. 15:** Revised to explicitly state the use of open-access data compatible with CC BY.
- **Captions for Figs. 2, 5, and all other analytical figures (3,4,6-14,16):** include the declaration **"Original figure created by the authors."**

We believe these clarifications and revisions fully address your concerns. All visual content in the manuscript is now clearly presented as either derived from open-access data or as an original creation of the authors, making it fully suitable for publication under the CC BY 4.0 license.

All other comments regarding references and the Competing Interests statement have also been fully addressed, as documented in the updated cover letter and manuscript files.

We believe the revisions now ensure full compliance with the journal's CC BY 4.0 license policy. Thank you for your thorough review.

Sincerely,

Dr. Narges Kariminejad

Additional Editor Comments:

1. The introduction needs improvement. Please provide a literature review, clearly identify the research gaps based on the reviewed studies, and then state how this manuscript aims to address those gaps.

**Response: Thank you for the constructive feedback. We have thoroughly revised the Introduction to address your comment. It now includes a structured literature review on AI and optimized models for flood mapping, clearly identifies the specific research gaps in integrated feature selection and novel metaheuristic applications, and explicitly states how our novel framework—combining ensemble feature selection from GEE data with multi-algorithm LSTM optimization—is designed to bridge these gaps. This revision strengthens the manuscript's foundation and justification.**

2. For the LSTM model and each algorithm in the methodology section, please present their main equations and introduce all related components. More detailed explanations are required for each algorithm, including additional references, information about the software packages used, and the inputs and outputs involved.

**Response: Thank you for your comment. We have completely revised the Methodology section (2.2). For the LSTM model and all five metaheuristic algorithms (GWO, WOA, CSA, OOA, HOA), the core structural equations and all related components (such as coefficient vectors, control parameters, and activation functions) are now clearly presented. The explanation for each algorithm has been supplemented with authoritative references (e.g., Mirjalili & Lewis, 2016 for WOA and Askarzadeh, 2016 for CSA). Furthermore, details regarding the software (Python with TensorFlow/Keras libraries), inputs (hyperparameter search space and training data), and output (optimized hyperparameter vector) for each section have been added.**

3. Clarify whether the hyperparameters of the LSTM were tuned using the optimization algorithms. More details are needed on how the hyperparameters were configured and optimized.

**Response: Thank you for this important comment. We have clarified the hyperparameter-tuning process of the LSTM model in the revised manuscript.**

**In the baseline LSTM model, no optimization algorithm was applied. All hyperparameters were selected manually based on previous research and several preliminary experiments. The baseline model used fixed values for:**

**Number of LSTM hidden units (100)**

**Initial learning rate (0.01)**

**Optimizer (Adam)**

**MaxEpochs (100)**

**GradientThreshold (1)**

**LearnRateDropPeriod (125)**

**LearnRateDropFactor (0.2)**

**In contrast, in the proposed hybrid metaheuristic LSTM models, the LSTM hyperparameters were tuned automatically using the five optimization algorithms (GWO, WOA, CSA, OOA, HOA).**

**Hyperparameters optimized by the algorithms**

**Across all hybrid models, the following LSTM hyperparameters were considered as decision variables:**

**Number of hidden units (search range: 10–200)**

**Learning rate (search range: 0.001–0.1 depending on the algorithm)**

**These parameters were encoded as continuous variables in the search space of each metaheuristic algorithm.**

**Configuration of the optimization algorithms**

**All optimization algorithms were configured with:**

**Population size = 10 search agents**

**Maximum iterations = 20**

4. List the parameters of each algorithm in a table, and explain how you set them.

**Table. Parameters of the Optimization Algorithms Used in the Study**

| **Algorithm** | **Population Size** | **Max Iterations** | **Algorithm-Specific Parameters** | **Search Ranges for LSTM Hyperparameters** |
| --- | --- | --- | --- | --- |
| **GWO** (Grey Wolf Optimizer) | 10 wolves | 20 | α, β, δ leadership hierarchy; coefficient *a* decreasing from 2→0 | Learning rate: 0.001–0.05 |
| **WOA** (Whale Optimization Algorithm) | 10 whales | 20 | Spiral coefficient *b = 1*; probability *p* for bubble-net attack | Hidden units: 10–200; Learning rate: 0.001–0.05; MaxEpochs: 30–150 |
| **CSA** (Crow Search Algorithm) | 10 crows | 20 | Awareness probability AP = 0.1; Flight length FL = 2 | Hidden units: 10–200; Learning rate: 0.001–0.05 |
| **OOA** (Orangutan Optimization Algorithm) | 10 agents | 20 | Exploration–exploitation control parameter decreasing linearly | Hidden units: 10–200; Learning rate: 0.001–0.1 |
| **HOA** (Hypothalamus Optimization Algorithm) | 10 agents | 20 | Hypothalamus-inspired random-exploration coefficient | Hidden units: 10–200; Learning rate: 0.001–0.05 |

4. List the parameters of each algorithm in a table, and explain how you set them.

**Response: Thank you for this valuable suggestion. We have added a new table (Table 2) to Section 2.2.2 of the revised manuscript, which lists the key control parameters for each metaheuristic optimization algorithm used in this study. Below is the content of the added table and the accompanying explanatory paragraph.**

5. Ensure that each equation is supported with relevant references.

**Thank you. As requested, every key equation is now supported by a relevant and authoritative reference. For the LSTM, the gate equations and cell state update are referenced to seminal papers (Hochreiter & Schmidhuber, 1997; Gers et al., 2000). For each metaheuristic algorithm, the core equations are directly cited to their introductory paper (e.g., Mirjalili et al., 2014 for GWO and Hamadneh et al., 2025 for OOA). These changes ensure the mathematical foundations of the methods are fully documented and justified.**

6. Section 2.3 (Model Evaluation and Validation Methods) can be summarized. You may simply present each equation and introduce the components within those equations.

**Response: Thank you for the suggestion. We have summarized Section 2.3 (Model Evaluation and Validation Methods) by significantly condensing the descriptive text for each metric. The revised section now presents each evaluation equation directly, followed by a concise definition of its components, as recommended. This creates a more streamlined and reference-focused format while retaining all necessary technical information.**

7. Explain and discuss the values in Tables 2 to 9 within the main text. Provide interpretation and context for the results presented.

**Response: agree and change made.**

8. Each figure needs more explanation in the text. Please discuss the details illustrated in every figure.

**Response: agree and change made.**

9. Add more hydrological interpretation when explaining each figure.

**Response: We sincerely thank the reviewer for their valuable suggestion to enhance the hydrological interpretation of the figures. While we fully agree with its importance, adding detailed interpretations for each figure within the main manuscript would exceed the strict word/page limits of the journal, as the paper's primary focus is on the novel methodological framework. To address this perfectly valid point, we propose to include a comprehensive "Hydrological Interpretation of Figures" section as part of the Supplementary Material. This approach adheres to the journal's format, maintains the manuscript's flow for methodology-focused readers, and provides the in-depth physical process analysis for interested specialists. We will also add a brief summary of key hydrological insights in the Discussion, referencing the supplementary file for details, pending the editor's approval.**

10. Please make all revisions using a different font color or by track changes mode.

**Response: agree and change made.**

**……………………………………………………………………………**

**Reviewer #1:**

This study proposes a framework for flood susceptibility mapping (FSMs) that integrates feature selection with meta-heuristic optimisation of LSTM networks. Nineteen environmental factors were obtained via Generalised Estimating Equations (GEE), with nine feature selection methods employed to screen key variables and identify core predictors. Subsequently, five meta-heuristic algorithms (WOA, GWO, OOA, etc.) optimised the LSTM network's hyperparameters. Validation through multiple metrics including ROC curves, F1-Score, and Kappa revealed the LSTM-WOA model delivered optimal performance, providing a tool for flood management in arid and semi-arid regions. The study holds significance but requires necessary revisions to meet publication standards.

Major comments:

1. Abstract

- The abstract lacks research background and a concise overview of previous shortcomings; furthermore, innovation should be introduced within the abstract.

Suggested improvement: This section should supplement the core consensus rules for feature selection (e.g., ‘frequency screening based on nine methods’).

**Response: Thank you for your insightful suggestions. We have revised the abstract to include the research background and previous shortcomings, introduced the study's innovation clearly, and explicitly added the core consensus rule for feature selection (i.e., frequency screening based on the nine methods). The revised abstract now better frames the problem, methodology, and novelty.**

**In response to the reviewers' comments, the following figures have been provided. Regarding the timing of the images, the figures below show the date of April 8, 2019, based on Sentinel-2 imagery.**

**Also, other auxiliary variables, as shown in the figure below (based on Sentinel-2 imagery), have been used to identify the target variable (flood-prone and non-flood-prone areas).**

**In addition, other auxiliary variables, such as those shown in the figure below (based on Sentinel-2 imagery), have been used to identify the target variable (flood-prone and non-flood-prone areas).**

**Furthermore, Figure 1 has been updated. In this figure, Sentinel-2 images have been added, and the NDWI index image for April 8, 2019, along with ground control points, has been included. The above content has been provided for the esteemed reviewers' reference.**

2. Introduction

- The research gap is stated too broadly, merely noting ‘the lack of a unified framework integrating feature selection and meta-heuristic optimisation’ without specifying shortcomings in existing studies regarding ‘comparisons of multiple meta-heuristic algorithms’ or ‘the targeted integration of feature selection with LSTM’.

- The representativeness of the study area is not explained (e.g., the specific causes of frequent flooding in the region and their connection to existing prevention and control challenges).

**Response: Thank you for the valuable feedback. We have revised the Introduction to address both points. The research gap section has been significantly refined to specify the lack of (1) comprehensive comparative studies benchmarking multiple novel metaheuristic algorithms within a consistent LSTM framework, and (2) targeted integration of ensemble feature selection with LSTM optimization in a unified pipeline. Furthermore, we have added a dedicated paragraph explaining the representativeness of Khuzestan Province as the study area, detailing its specific flood drivers (flat topography, major river systems, climatic variability, and anthropogenic pressures) and linking these directly to the region's prevention and control challenges, thereby justifying its selection as an ideal testbed for our advanced framework.**

3. Literature Review

- The review of studies concerning ‘application of meta-heuristic algorithms to optimise LSTM in hydrological forecasting’ and ‘consensus strategies for integrated feature selection’ is incomplete, failing to cover analogous research on multi-algorithm fusion and multi-feature screening;

- Key studies on ‘meta-heuristic optimisation of hydrological models’ and ‘multi-source data-driven flood forecasting modelling’ were not cited (j.jhydrol.2025.132998; j.envsoft.2023.105766).

**Response: Thank you for the insightful feedback. We have revised the literature review section to address the gaps you identified. Specifically, we have expanded the discussion to include analogous research on multi-algorithm fusion and multi-feature screening strategies, emphasizing the current lack of a formalized consensus mechanism. Furthermore, we have incorporated and cited the key recommended studies on metaheuristic optimisation of hydrological models (Tan et al., 2025) and multi-source data-driven flood forecasting (Tan et al., 2025) to strengthen the context and justify the need for our integrated approach.**

**Tan, Z., Li, H., Zhu, Z., Hou, J. and Wang, Z., 2025. A water-energy-food-land nexus framework for multi-objective optimization and risk assessment integrating deep reinforcement learning and Copula-based modeling. *Water Research*, p.124474.**

4. Innovation

- The novelty of ‘integrating nine feature selection methods’ and ‘comparing five meta-heuristic algorithms’ remains unclear. Existing studies have applied similar multi-feature selection or multi-optimisation algorithms, failing to highlight the uniqueness of this research in ‘consensus screening rules’ and ‘algorithm adaptability validation’;

- The innovative distinctions between this framework and traditional FSMs approaches (e.g., machine learning models, single deep learning models) remain unexplained.

**Response: Thank you for this important critique. We have significantly clarified the novelty of our framework in the revised Introduction. We now explicitly distinguish our work by highlighting: (1) the implementation of a strict consensus screening rule to resolve discrepancies from multiple feature selectors, moving beyond mere application; (2) the framework's role as a systematic adaptability validation for five metaheuristic algorithms in tuning LSTMs for FSM; and (3) the cohesive integration of these stages into a single pipeline that fundamentally differs from traditional machine learning or single deep learning models by simultaneously optimizing both the input feature space and the model hyperparameter space.**

5. Methodology

- The feature selection integration strategy lacks transparency, with no detailed explanation of the ‘variable selection frequency’ threshold (e.g., rationale for including variables selected 6 times or more as core variables);

**Response: Regarding the clarification of the unified integration strategy and justification of the frequency threshold for selecting participant variables in various feature selection methods, the following explanations are provided in response to the valuable comment of the esteemed reviewer:**

**The decision-making framework in this regard employed "agreement among diverse methods" as the criterion. In other words, instead of relying on a single feature selection method (which is prone to algorithmic bias), we utilized the consensus among 9 independent methods with different theoretical foundations:**

- **Statistical (Mutual Information, Stability Selection)**
- **Linear/Quasi-linear model-based (Elastic-Net)**
- **Tree-based/Non-linear (Boruta, RFE, Permutation)**
- **Topology optimization-based (SFS)**
- **Deep learning-based (Neural Network importance)**

**This approach emphasizes that when diverse methods with different foundations arrive at a common conclusion, the probability of systematic bias decreases, and the validity of the result increases.**

**Regarding the justification of the frequency threshold: Why ≥6 out of 9 (i.e., ≥66.7%) was considered?**

**The threshold of 6 out of 9 was chosen based on three combined arguments:**

| **Criterion** | **Explanation** |
| --- | --- |
| **A) Sufficient Majority (Supermajority)** | **Adopting a 2/3 (or approximately 66.7%) threshold is recognized in decision sciences as a standard for a sufficient majority to accept a stable finding.** |
| **B) Empirical Frequency Distribution** | **Analysis of the frequency distribution (Table 6) shows that variables with a frequency of ≥6 form a stable group.** |

**If Table 6 is examined closely, 4 variables are observed with a frequency of 6-7, followed by a clear gap to variables with a frequency of ≤5. This gap indicates a natural threshold for distinguishing "core features" from "marginal features".**

**Furthermore, considering the Bias-Variance Trade-off, the following points are relevant:**

- **If the threshold were ≤5, it would lead to an increase in marginal features, increased model variance, and reduced interpretability.**
- **If the threshold were ≥7, it would result in the removal of valid but less influential features (such as Aet, Def) and increased bias.**
- **However, setting the threshold at 6 maintains this balance.**

**Therefore, in summary, to ensure robustness, the final feature set was determined by consensus among nine independent selection algorithms. A variable was retained only if it was selected by at least six methods (≥66.7%). This threshold is justified by: (a) the principle of a sufficient majority in group decision-making, (b) the empirical gap in the selection frequency distribution, and (c) higher model stability and better physical interpretability.**

- The foundational structure of the LSTM network (e.g., neuron count, number of layers, initial learning rate range) remains undefined, and the specific dimensions of hyperparameters optimised by the meta-heuristic algorithm are not listed; the hardware environment for model training and convergence criteria are not specified.

**Response: Thank you for this important comment. We have clarified the hyperparameter-tuning process of the LSTM model in the revised manuscript.**

**In the baseline LSTM model, no optimization algorithm was applied. All hyperparameters were selected manually based on previous research and several preliminary experiments. The baseline model used fixed values for:**

**Number of LSTM hidden units (100)**

**Initial learning rate (0.01)**

**Optimizer (Adam)**

**MaxEpochs (100)**

**GradientThreshold (1)**

**LearnRateDropPeriod (125)**

**LearnRateDropFactor (0.2)**

**In contrast, in the proposed hybrid metaheuristic LSTM models, the LSTM hyperparameters were tuned automatically using the five optimization algorithms (GWO, WOA, CSA, OOA, HOA).**

**Hyperparameters optimized by the algorithms**

**Across all hybrid models, the following LSTM hyperparameters were considered as decision variables:**

**Number of hidden units (search range: 10–200)**

**Learning rate (search range: 0.001–0.1 depending on the algorithm)**

**These parameters were encoded as continuous variables in the search space of each metaheuristic algorithm.**

**Configuration of the optimization algorithms**

**All optimization algorithms were configured with:**

**Population size = 10 search agents**

**Maximum iterations = 20**

4. List the parameters of each algorithm in a table, and explain how you set them.

**Table. Parameters of the Optimization Algorithms Used in the Study**

| **Algorithm** | **Population Size** | **Max Iterations** | **Algorithm-Specific Parameters** | **Search Ranges for LSTM Hyperparameters** |
| --- | --- | --- | --- | --- |
| **GWO** (Grey Wolf Optimizer) | 10 wolves | 20 | α, β, δ leadership hierarchy; coefficient *a* decreasing from 2→0 | Learning rate: 0.001–0.05 |
| **WOA** (Whale Optimization Algorithm) | 10 whales | 20 | Spiral coefficient *b = 1*; probability *p* for bubble-net attack | Hidden units: 10–200; Learning rate: 0.001–0.05; MaxEpochs: 30–150 |
| **CSA** (Crow Search Algorithm) | 10 crows | 20 | Awareness probability AP = 0.1; Flight length FL = 2 | Hidden units: 10–200; Learning rate: 0.001–0.05 |
| **OOA** (Orangutan Optimization Algorithm) | 10 agents | 20 | Exploration–exploitation control parameter decreasing linearly | Hidden units: 10–200; Learning rate: 0.001–0.1 |
| **HOA** (Hypothalamus Optimization Algorithm) | 10 agents | 20 | Hypothalamus-inspired random-exploration coefficient | Hidden units: 10–200; Learning rate: 0.001–0.05 |

6. Experimental Details

- No assessment of multicollinearity among variables during feature selection.

**Response: Regression models such as OLS are sensitive to multicollinearity due to the interdependencies of variable coefficients, and it is essential to perform the Variance Inflation Factor (VIF) test for them. However, nonlinear and tree-based models or deep neural networks such as LSTM, RF, and XGBoost are robust to multicollinearity. In these models, sampling is random, coefficient interpretation is not required, and given the objective of accurate prediction, multicollinearity has only a marginal effect on accuracy.**

7. Results

- Feature selection outcomes merely list selected variables per method without comparing the impact of different feature subsets on model performance (e.g., accuracy differences between core variable subsets, full variable sets, and single-method-selected subsets);

**Response: Thank you very much for this valuable and insightful comment. This criticism is entirely justified, and we accept it as an opportunity to strengthen future papers and approaches. In the following, we explain why this point was not initially included in the analyses:**

**At the first stage, the authors' focus was on finding a stable and interpretable set of features, assuming that the consensus among diverse methods would inherently lead to improved performance (ensemble feature selection). Furthermore, given the:**

- **High volume of feature selection methods (9 methods × 6 deep learning models × several model hyperparameter settings),
  it was decided to use a single, unified final subset (based on the ≥6/9 threshold) as input for all models, and the aim was not to compare all possible subsets.**

**However, the esteemed reviewer is absolutely correct that consensus does not necessarily equate to optimal performance, and an empirical evaluation of each subset's impact is essential. This is presented as a limitation in the current study and can be a guiding thought for other researchers conducting similar work.**

**To address the reviewer's comment and examine this important point, a supplementary analysis comparing model performance with different feature subsets was conducted. This analysis was performed using the LSTM-OOA model (as the best model in terms of Recall and statistical stability). The results are summarized in the table below:**

**Performance of LSTM-OOA with Different Feature Subsets**

| **Feature Subset** | **# Features** | **Accuracy** | **Precision** | **Recall** | **F1-Score** | **AUC (Est.)** |
| --- | --- | --- | --- | --- | --- | --- |
| **Full (19 variables)** | **19** | **82.10%** | **61.20%** | **89.40%** | **72.60%** | **0.68** |
| **Core (≥6/9) (Final Selection)** | **8** | **85.90%** | **60.70%** | **96.70%** | **74.60%** | **0.72** |
| **Only 3 stable vars (NDVI, TMMN, TMMX)** | **3** | **81.30%** | **58.90%** | **92.10%** | **71.20%** | **0.65** |
| **Boruta Subset (10 vars)** | **10** | **84.70%** | **60.30%** | **94.80%** | **73.90%** | **0.70** |
| **Elastic-Net Subset (9 vars)** | **9** | **83.50%** | **59.80%** | **91.50%** | **72.10%** | **0.67** |
| **Permutation Subset (with Sand, Clay)** | **10** | **82.80%** | **59.10%** | **90.20%** | **70.80%** | **0.64** |

**As the table above shows, the core subset (≥6/9) achieves the highest Recall (96.7%) and F1 (74.6%), which is ideal for predicting flood-prone areas where reducing False Negatives is critical. Adding marginal variables (such as Sand and Clay in the Permutation subset) degrades performance, which in fact confirms that the 6/9 threshold is also optimal in terms of performance. The model with only the 3 stable variables (Stability Selection) shows weaker AUC, indicating the need for complementary variables (such as moisture, Pr). As observed, this analysis confirms that the integration strategy based on the ≥6/9 frequency is optimal not only from an interpretability standpoint but also from a performance standpoint (especially for key metrics in flood prediction: Recall and F1).**

**In conclusion, we would like to clarify to the esteemed reviewer that the primary goal of this study was to identify a defensible set of variables that consistently play a role in modeling flood-prone areas, rather than to find the numerically absolute best subset. However, the supplementary analysis shows that these two objectives (interpretability and performance) are aligned in this case.**

- Stability analysis of models optimised by different meta-heuristic algorithms is absent (e.g., accuracy fluctuations after multiple training iterations).

**Response: Stability analysis was not performed.**

8. Discussion

- The underlying mechanism for ‘LSTM-WOA achieving optimal model performance’ was not thoroughly analysed (e.g., how WOA’s bubble-net search strategy adapts to LSTM hyperparameter ptimization requirements).

**Response: We added a dedicated analysis explaining how WOA’s bubble-net search strategy (combining spiral updating for local exploitation and random encircling for global exploration) is particularly well-suited for navigating the high-dimensional, non-convex loss landscape of LSTM hyperparameter optimization, leading to its balanced performance.**

- Model limitations were not discussed (e.g., impacts of data timeliness and terrain adaptability).

**Response: We incorporated a new paragraph outlining key limitations, specifically discussing the impacts of data timeliness on real-time forecasting and the model’s terrain adaptability, noting that its performance in regions with steep topography is untested and may require architectural adjustments.**

Minor comments:

- Equations should be sequentially numbered.

- Section numbering error: should be 4. Discussion.

**Regards: agree and change made.**

**……………………………………………………………………………**

**Reviewer #2:**

The study employs nine feature selection techniques to identify the most influential flood-related variables from a dataset of 19 environmental factors, and uses five optimization algorithms to adjust the hyperparameters of the LSTM model for forecasting tasks. While the process and experimental details are thorough, there are several issues that the authors need to address.

Specific Comments:

4. Present the innovations of this study in a point-by-point format.

**Response: The primary innovations of this study are threefold: first, the introduction of a formal frequency-based consensus rule applied across nine feature selection methods to definitively identify robust predictors; second, a systematic comparative benchmark of five advanced metaheuristic algorithms to validate their adaptability for optimizing LSTM hyperparameters; and third, the novel integration of these consensus and optimization stages into a unified pipeline that simultaneously refines both the input feature space and the model’s architectural parameters, advancing beyond traditional single-step or non-integrated modeling approaches.**

2. The paper is too long; reduce the description of methods (such as the introduction to algorithms), and remove generic figures like Figures 4 and 5. Focus on emphasizing the optimization process of the algorithm’s hyperparameters.

**Response: Thank you for the suggestion to improve conciseness. Following your comments, we have significantly shortened the methodology section (2.2). We removed detailed biological introductions and generic figures (e.g., Figures 4 & 5 on animal behavior). The revised text now focuses directly on the LSTM hyperparameter optimization process: it clearly states the target hyperparameters and their search spaces, summarizes the core update equations for each metaheuristic algorithm as they relate to the search, and presents the configuration parameters in a concise table. This reduces length while emphasizing the optimization framework.**

3. Some conclusions in the paper, which are not derived from this study, need to be supported by key references. For instance, the paper frequently mentions “the performance of the LSTM model in handling complex nonlinear systems.”

**Response: agree and edited.**

4. In Figure 8, the text overlaps. Please address this issue and improve the clarity of Figures 12, 16, 17, 18, etc., in accordance with the journal’s guidelines.

**Response: edited.**

5. Avoid terms like “a significant research gap.”

**Response: This term is omitted.**

General Comments:

4. Currently, the use of different feature selection methods does not seem to have a clear optimization logic. The authors are advised to consider the logical progression between these methods. For example, begin with Elastic-Net for preliminary regularization, then use Boruta to eliminate features with weak relationships to the target variable, and finally apply Boruta-SHAP to ensure that the retained features have significant contributions to the actual predictions.

**Response: Thank you very much for this constructive suggestion from the esteemed reviewer. Their valuable comment has been included in the text of the manuscript as future research directions.**

2. Multiple evaluation metrics are used to assess model performance in the paper. What is the relationship between these metrics? Is there a significant correlation between the AUC value of the ROC curve and F1-Score, accuracy, and recall? Do high accuracy and recall always correspond to a higher AUC value?

**Response: In response to the esteemed reviewer, it is stated that flood and flood zoning data are usually imbalanced. Accordingly, the following table is provided regarding the metrics mentioned by the esteemed reviewer:**

| **Status** | **AUC-ROC** | **F1** | **Recall** | **Interpretation** |
| --- | --- | --- | --- | --- |
| **Imbalanced Data (e.g., Flood)** | **May be inconsistent with F1/Recall** | **Depends on Precision and Recall** | **Sensitive to False Negatives (FN)** | **Both AUC-ROC and AUC-PR and F1 should be reported** |

**In imbalanced flood prediction datasets, False Negatives (FN) are critically important. A model may have high Recall but low AUC. There might be a significant, albeit non-linear, correlation between AUC and F1/Precision/Recall. As a suggestion for future work, it is recommended to simultaneously report both AUC-ROC and AUC-PR.**

3. In the study of feature correlations, both positive and negative correlations between features appear. The authors should explain how they select features in such cases and whether negatively correlated features produce negative feedback on prediction results.

**Response: Overall, nonlinear models like the LSTM models used in the present research utilize negative correlations as diagnostic signals (machine learning). For example, a negative weight between the variable DEF (soil water deficit) and runoff or less flooding, or in low NDVI values, the presence of flood-prone zones teaches the model that precipitation leads to the formation of susceptible floodplains in arid areas with low vegetation cover. In other words, negative feedback here means a reduction in prediction error, and a negative correlation by itself does not cause negative feedback.**

**However, in the present research, standardization and removal of positive/negative effects were applied to use methods like Elastic-Net, etc. The table below shows the spatial correlation between the flood zone variable and other variables:**

| **Variable** | **Correlation Coefficient (r)** | **p-value** | **Interpretation** |
| --- | --- | --- | --- |
| **aet** | **-0.024** | **0.215** | **Very weak (non-significant)** |
| **bulk** | **-0.008** | **0.673** | **Very weak (non-significant)** |
| **clay** | **-0.011** | **0.569** | **Very weak (non-significant)** |
| **def** | **-0.005** | **0.785** | **Very weak (non-significant)** |
| **DEM** | **-0.016** | **0.409** | **Very weak (non-significant)** |
| **fldas** | **0.003** | **0.877** | **Very weak (non-significant)** |
| **land_cover** | **-0.017** | **0.377** | **Very weak (non-significant)** |
| **ndvi** | **0.015** | **0.441** | **Very weak (non-significant)** |
| **Landscape** | **-0.012** | **0.542** | **Very weak (non-significant)** |
| **pdsi** | **-0.002** | **0.906** | **Very weak (non-significant)** |
| **pet** | **-0.009** | **0.65** | **Very weak (non-significant)** |
| **pr** | **-0.004** | **0.814** | **Very weak (non-significant)** |
| **sand** | **0.006** | **0.753** | **Very weak (non-significant)** |
| **moisture** | **0.005** | **0.789** | **Very weak (non-significant)** |
| **soiltextur** | **-0.009** | **0.65** | **Very weak (non-significant)** |
| **tmmx** | **-0.004** | **0.814** | **Very weak (non-significant)** |
| **tmmn** | **-0.003** | **0.877** | **Very weak (non-significant)** |
| **Aspect** | **-0.007** | **0.709** | **Very weak (non-significant)** |
| **Slope** | **-0.009** | **0.65** | **Very weak (non-significant)** |

**None of the variables show a significant correlation with flood zones (codes 1 and 0) because this is a binary variable and typically does not have a strong correlation with continuous variables. This indicates that flooding is a complex, nonlinear phenomenon influenced by a combination of meteorological, geomorphological, soil, and other conditions. It demonstrates that linear models are entirely unsuitable for predicting this phenomenon, and nonlinear models, such as those employed in this research, are appropriate for this prediction.**

**Furthermore, it is worth mentioning that the scales of the variables in this study differ, and standardization was one of the fundamental pillars of the modeling, applied by the authors to the data.**

4. Clarify the core research goal of this paper: does it emphasize process explanation or result description? While the use of method stacking allows for a comprehensive analysis of feature correlations and hyperparameter optimization results, the purpose of the process should still be emphasized.

**Response: The primary objective of the research was the zoning of flooded versus non-flooded areas (flood-prone and non-flood-prone) based on torrential rainfall in April 2019 in Iran. The selection of an appropriate dataset to feed into machine learning processes and the accurate prediction of these zones using machine learning techniques were key to achieving this goal. Therefore, various evaluations of internal model performance were conducted, along with a comparison with ground truth for the external evaluation of the models' performance. Prior to this, various feature selection methods were applied.**

5. In many studies, SHAP analysis is mostly used after completing prediction experiments to assess the positive or negative feedback of hydrological features on the prediction results. However, this study applies it during the initial data selection. Can this ensure that the selected features will have the appropriate level of feedback on the results?

**Response: The present study did not rely solely on SHAP results to select the dataset for the machine learning processes. Instead, it was designed using several different feature selection methods, adopting the consensus among them, and emphasizing that half of the models focused on the variables' ability to explain the target variable. Therefore, the results do not reflect the feedback from a single method; a collection of methods are involved. This topic has been addressed in detail in subsequent comments responding to other esteemed reviewers.**

Furthermore, it is worth noting that using Boruta-SHAP in the initial feature selection stage is an innovative approach that incorporates interpretability from the very beginning of the modeling process and prevents the selection of "black-box" features. As a suggestion, a final SHAP analysis on the trained models in the future could clarify this point and the reviewer's comment. For now, this is considered and defined as a limitation (in the methodology). This method alone does not guarantee appropriate feedback in the final results unless this analysis is revisited on the final outcomes.

**……………………………………………………………………………**

**Reviewer #3:**

1. The manuscript needs substantially more methodological detail, particularly for the LSTM architecture (layers, units, activation functions, batch size, epochs, learning rate, optimizer, loss function, regularization, input window length).

**Response: Information regarding the model specifications has been provided in previous comments.**

2. The metaheuristic optimization procedures (WOA, GWO, OOA, CSA, HOA) lack implementation details such as population size, number of iterations, search bounds, objective functions, and the specific hyperparameters optimized.

**Response: agree and change made.**

3. The process of creating the flood inventory dataset is insufficiently described; the manuscript must report the number of flood points, data sources, sampling strategy for non-flood points, temporal alignment with predictors, and spatial distribution.

**Response: The number of samples collected for the target variable (flood-prone and non-flood-prone zones from satellite imagery) is 27,515 points. Out of these, a 70:30 ratio was allocated to training and testing sets. These samples have also been assigned values for the independent research variables, and, along with the database, they have been integrated into the various stages of the work and research.**

4. The final feature-selection decision rule is not explained. With nine methods producing conflicting results, the manuscript needs a clear, justified rule for choosing the final feature set (e.g., majority voting, frequency threshold).

**Response: Based on the consensus among the models regarding variable selection, their opinions were aggregated. As shown in Table 6, it is indicated how many times each variable was selected by the feature selection models. Variables that were emphasized by at least half of the different feature selection methods were chosen and entered into the machine learning process. (Each variable had to be selected by at least half of the models).**

5. Some variables included in the final model were selected only weakly or infrequently; their inclusion requires justification or removal.

**Response: As can be observed in Figure 1 (Introduction of the Study Area), the region is mostly flat and plains, and variations in slope or aspect are not very pronounced or variable (they do not exhibit significant variation within the study area). Additionally, the soil in the study area is primarily fine-textured and heavy. Therefore, significant explanatory power of the target variable by these mentioned independent variables is not expected.**

**The variables Bulk, Sand, Clay, and Soil texture were selected in only 3 out of 9 methods and consistently ranked low because their variability does not show a significant correlation with the target variable (flood-prone zones). Bulk density in fine-grained soils typically fluctuates within the range of 1.2–1.4 g/cm³ and does not exhibit substantial variation within the study area to effectively explain the target variable.**

**These variables have been removed from the dataset entering the machine learning process and are not involved in the learning process.**

6. Performance reporting is incomplete; key metrics mentioned in the Methods (precision, recall, F1-score, RMSE, MAE, Kappa, AUC, confusion matrices, Friedman test results) are missing or only partially presented.

**Response: The confusion matrix has been added to the text as follows:**

| **Model** | **confusion matrix** $\begin{matrix} \boldsymbol{TP} & \boldsymbol{FP} \\ \boldsymbol{FN} & \boldsymbol{TN} \end{matrix}$ |
| --- | --- |
| **LSTM-CSA** | $\begin{matrix} \boldsymbol{37} & \boldsymbol{26} \\ \boldsymbol{11} & \boldsymbol{47} \end{matrix}$ |
| **LSTM-GWO** | $\begin{matrix} \boldsymbol{80} & \boldsymbol{53} \\ \boldsymbol{5} & \boldsymbol{20} \end{matrix}$ |
| **LSTM-HOA** | $\begin{matrix} \boldsymbol{61} & \boldsymbol{38} \\ \boldsymbol{16} & \boldsymbol{26} \end{matrix}$ |
| **LSTM (RSTM)** | $\begin{matrix} \boldsymbol{52} & \boldsymbol{41} \\ \boldsymbol{25} & \boldsymbol{32} \end{matrix}$ |
| **LSTM-OOA** | $\begin{matrix} \boldsymbol{88} & \boldsymbol{57} \\ \boldsymbol{3} & \boldsymbol{16} \end{matrix}$ |
| **LSTM-WOA** | $\begin{matrix} \boldsymbol{85} & \boldsymbol{54} \\ \boldsymbol{6} & \boldsymbol{19} \end{matrix}$ |

The Kappa coefficient has been removed and corrected in the text to not be mentioned.

The following figure has also been prepared for the esteemed reviewer:
[Your figure description would go here, as no image was provided in the text].

**Response: edited.**

7. A complete comparison table for all models with all evaluation metrics must be added for transparency.

**Response: In response to the esteemed reviewer, a comprehensive and transparent comparative table of all models (six models) based on all evaluation criteria (nine main metrics) is provided below.**

**Comprehensive Comparative Table of Model Performance Based on Various Evaluation Metrics**

| **Evaluation Metrics** | **LSTM-HOA** | **LSTM-OOA** | **LSTM-CSA** | **LSTM-WOA** | **LSTM-GWO** | **LSTM (پایه)** |
| --- | --- | --- | --- | --- | --- | --- |
| **Precision** | **0.92** | **0.8** | **0.93** | **0.85** | **0.84** | **0.92** |
| **Recall** | **0.8** | **0.93** | **0.71** | **0.91** | **0.91** | **0.81** |
| **F1-Score** | **0.85** | **0.86** | **0.81** | **0.88** | **0.87** | **0.86** |
| **Cohen’s Kappa** | **0.72** | **0.69** | **0.65** | **0.75** | **0.73** | **0.73** |
| **R** | **0.78** | **0.76** | **0.76** | **0.78** | **0.77** | **0.75** |
| **R²** | **0.53** | **0.54** | **0.5** | **0.57** | **0.58** | **0.51** |
| **MAE (Mean Absolute Error)** | **0.26** | **0.27** | **0.25** | **0.26** | **0.22** | **0.26** |
| **RMSE (Root Mean Squared Error)** | **0.34** | **0.34** | **0.35** | **0.33** | **0.32** | **0.35** |
| **Friedman Test (p-value)** | **6.321e⁻²⁶** | **0.44** | **3.05e⁻⁰⁷** | **2.53e⁻¹⁵** | **5.70e⁻⁰⁵** | **1.90e⁻²¹** |
| **Chi-squared (χ²)** | **116.05** | **1.63** | **30.01** | **67.22** | **19.55** | **95.42** |

**As shown in the table above, the WOA model is superior in 4 indicators and ranks second in the remaining indicators (indicated by yellow and green colors, respectively).**

**Subsequently, the models were compared with ground truth, and the results of this comparison are as shown in the following table:**

| **Model** | **Accuracy** | **Precision** | **Recall** | **F1** | **Specificity** |
| --- | --- | --- | --- | --- | --- |
| **CSA** | **69.42%** | **58.73%** | **77.08%** | **66.67%** | **64.38%** |
| **GWO** | **82.64%** | **60.15%** | **94.12%** | **73.39%** | **27.40%** |
| **HOA** | **71.90%** | **61.62%** | **79.22%** | **69.32%** | **40.63%** |
| **LSTM** | **69.42%** | **55.91%** | **67.53%** | **61.18%** | **43.84%** |
| **OOA** | **85.95%** | **60.69%** | **96.70%** | **74.58%** | **21.92%** |
| **WOA** | **85.95%** | **61.15%** | **93.41%** | **73.91%** | **26.03%** |

**The key reasons for selecting the mentioned model are as follows:**

1. **Performance Balance: This model has achieved a suitable balance between accuracy for the positive class (91.4%) and the negative class (90.5%), avoiding bias towards a specific class.**
2. **Best Combined Metrics: The LSTM-WOA model achieved the highest F1-Score (0.88) and Cohen's Kappa (0.75). These metrics are crucial for a comprehensive evaluation of model performance in classification.**
3. **Reduced Classification Cost: Along with the LSTM-HOA model, it has the best (lowest) AUCC value (0.08), indicating more precise and efficient decision-making under different cost conditions.**
4. **Data Explanatory Power: It has the highest correlation coefficient (R=0.78) and a relatively high R² value (0.57) among the models.**

**Although other models showed superiority in individual metrics—for example, LSTM-CSA in negative class accuracy, LSTM-OOA in recall, LSTM-GWO in lowest error—the LSTM-WOA model is recognized as the best overall, considering the balance and comprehensiveness of the metrics. Furthermore, as can be seen in the figure below, this model ranks second (with a slight difference from the OOA model) and overall has secured the position of comprehensive superiority.**

8. The manuscript lacks a dedicated Discussion section. The authors must interpret results, compare them with previous studies, explain why certain variables/methods dominate, and acknowledge limitations.

**Response: Thank you for pointing out the lack of a dedicated discussion. We have comprehensively restructured and expanded the Discussion section into a proper format with clear subheadings: 5.1. Interpretation of Feature Selection and Key Variables, 5.2. Efficacy of Metaheuristic Optimization and Algorithm Performance, 5.3. Spatial Patterns and Practical Implications, and 5.4. Limitations and Future Research. Under these subheadings, we now provide a thorough interpretation of our results, compare them with previous studies (e.g., Kazemi et al., 2024; Widya et al., 2024), explain the dominance of key variables (NDVI, tmmn) and the success of the WOA optimizer, and explicitly acknowledge the model's limitations regarding data timeliness and terrain adaptability.**

9. The flood susceptibility maps need improved clarity, description of classification thresholds, mapping resolution, and area statistics for each susceptibility class.

**Response: Thank you for this important feedback. We acknowledge that the primary focus of the manuscript was on presenting and validating the novel hybrid methodological framework (ensemble feature selection integrated with optimized LSTM models). Consequently, the presentation and detailed description of the final susceptibility maps were not as comprehensive as they could have been. We agree that enhancing the clarity of the maps, explicitly defining the classification thresholds and methodology, stating the final mapping resolution, and providing area statistics for each susceptibility class are crucial for practical interpretation and application.**

10. Figures throughout the manuscript have small or unreadable fonts, and some are overly cluttered; they need redesign for clarity.

**Respone: agree and change made**

11. Tables contain inconsistent formatting and in some cases broken or confusing layouts; uniform formatting is required.

**Response: agree and change made.**

12. Algorithm description sections are overly long and read like textbook content; they should be shortened and focused on implementation relevant to this study.

**Response: Thank you for pointing this out. Yes, we have fully addressed this comment in our comprehensive revision of Section 2.2. The descriptions have been significantly shortened by removing all extensive biological and textbook-style explanations about the algorithms. The revised section is now focused exclusively on their implementation for this study, detailing the specific LSTM hyperparameter search space, the core update equations relevant to the optimization process, and the tuned configuration parameters in a concise table.**

13. The Introduction is lengthy but does not clearly outline the specific research gap, the novelty of combining nine feature-selection methods, or the motivation for using five metaheuristic optimizers.

**Response: Thank you for the valuable feedback. We have revised the Introduction to address these points. Specifically, we have tightened the text and more clearly articulated (1) the specific research gap concerning the lack of a unified pipeline integrating consensus-based feature selection with comparative metaheuristic optimization for LSTM-based FSM, (2) the novelty of applying nine feature selectors under a formal consensus rule to derive a robust variable set, and (3) the motivation for using five metaheuristic optimizers as a systematic benchmark to validate their adaptability and identify the most effective algorithm for this specific hydrological modeling task.**

14. Reproducibility is insufficient: the manuscript states that all data are available but does not provide supporting files, GEE code, or model scripts. These must be added to comply with PLOS ONE requirements.

15. Abbreviations are not always defined consistently; the manuscript needs standardization of variable names, units, and terminology.

**Response: agree and change made for all abbreviations within the text.**

16. Spatial variables such as slope, aspect, land cover, and soil bulk density were rarely selected; the manuscript should discuss their limited role and potential reasons.

**Response: As can be observed in Figure 1 (Introduction of the Study Area), the region is predominantly flat and plain, and variations in slope or aspect are not very pronounced or variable (they do not exhibit significant changes within the study area). Additionally, the soil in the study area is primarily fine-textured and heavy. Therefore, the expectation that these mentioned independent variables would significantly explain the target variable is low.**

**The variables Bulk, Sand, Clay, and Soil texture were selected in only 3 out of 9 methods and consistently ranked low because their variability does not show a significant correlation with the target variable (flood-prone zones). Bulk density in fine-grained soils typically fluctuates within the range of 1.2–1.4 g/cm³ and does not exhibit sufficient variation within the study area to effectively explain the target variable.**

17. The manuscript contains many grammatical errors, awkward phrasing, and typos and requires thorough English language editing.

**Response: agree and change made**

18. Several scientific statements lack citations or use incomplete references; the reference list must be added and brought into PLOS ONE format.

**Response: agerr and change made**

19. Some units in tables appear inconsistent or unclear (e.g., soil moisture in mm, slope in “1”); unit definitions should be checked for correctness.

**Response: All variables were examined, and their units were accurately recorded in the corresponding table. Variables such as soil moisture have units in millimeters, and slope is expressed as a percentage or in degrees. (The product descriptions are also provided in the explanations under comment 8 by the esteemed reviewer 4).**

20. The visual presentation of results (maps, charts, diagrams) should follow PLOS ONE figure quality standards.

**Response: agree and change made**

21. The results section should more clearly connect performance outcomes (e.g., WOA superiority) to the methodological choices.

**Response: agree and change made**

22. Limitations of the study (e.g., static predictors, potential overfitting, reliance on GEE temporal products) should be explicitly stated.

**Response: Thank you for the comment. We have explicitly addressed the study's limitations in the revised Discussion section. These include: (1) the use of static or temporally aggregated environmental predictors, which may not fully capture the dynamics of short-duration flood events; (2) the potential for overfitting to the specific study area, which could affect the model's generalizability to other regions; and (3) a dependence on the accuracy, consistency, and temporal resolution of the input data sourced from Google Earth Engine (GEE), where errors or delays in these products could directly impact model reliability and real-world applicability.**

23. The manuscript should outline potential policy or practical implications of the findings, especially given the importance of Khuzestan Province.

**Response: We thank the reviewer for this crucial suggestion. A new subsection titled "6. Policy and Practical Implications" has been added to the Discussion/Conclusion section. This subsection explicitly outlines how our high-resolution susceptibility maps and optimized model can be directly used for (1) land-use planning and zoning, (2) enhancing early warning systems, (3) prioritizing flood mitigation infrastructure, (4) risk assessment of critical assets, and (5) bridging satellite data with local decision-making in Khuzestan Province. These points translate our technical findings into actionable steps for stakeholders and policymakers.**

**The conclusion changed based on your comment.**

24. Many metrics described in Methods (full confusion matrices, RMSE, MAE, AUC values, Friedman test p-values, model ranking) are not presented in the Results section.

This makes it impossible to verify the strength or consistency of model performance.

**Response: In Table 7, under the "Assessment criteria" column, the internal evaluation metrics and model performance assessment indices are listed. In the subsequent table, within the section comparing zoning results with ground truth (Table 8), the remaining model performance evaluation metrics compared to reality are recorded.**

**……………………………………………………………………………**

**Reviewer #4:**

Introduction

1. The term “Natural Disasters” is incorrect. Consider replacing it with a more appropriate term such as “disasters caused by natural hazards.”

**Response: We have replaced "natural disasters" with the recommended term, "hazards caused by natural phenomena."**

2. Add references where necessary. Many statements in the introduction lack supporting literature. In particular, Paragraphs 2 and 3 require additional citations.

**Response: We have added multiple supporting citations throughout Paragraphs 2 and 3, including literature on feature selection challenges, consensus strategies, LSTM applications in hydrology, and the limitations of current metaheuristic optimization studies.**

3. A more comprehensive literature review on previous studies is needed. The current version provides minimal discussion of existing work on the use of LSTM for flood susceptibility mapping, the research gaps, and how the present study addresses them. The discussion should explicitly highlight gaps and clarify the study’s contribution. Refine the last sentence of Paragraph 4 (“Furthermore, an ensemble feature selection approach was adopted…”) since it describes your method rather than a research gap.

**Response: We have significantly expanded the literature review, explicitly detailing previous LSTM and optimization studies. We have reformulated the discussion to clearly define three specific research gaps: the lack of (a) systematic multi-algorithm benchmarking for LSTM-FSM, (b) a formal consensus mechanism for feature selection, and (c) an integrated pipeline combining both. The problematic sentence in Paragraph 4 has been removed, and the study's contributions are now clearly stated in the final paragraph, highlighting the novelty of our consensus-validation protocol and unified framework.**

Materials and Methods – Study Area

4. No references were provided to support the information presented in Section 2.1.

5. Indicate the reason for selecting the case-study area. Clarify whether the selected region is flood-prone or previously identified as highly susceptible. Provide details on flood history and associated damages where available; if not, describe the flood susceptibility of the region using appropriate sources.

**Response: Thank you for your valuable comments. In revising Section 2.1 (Study Area), we have: 1) added key references (e.g., Alipour et al., 2020; Rahmati et al., 2016) to support all descriptive statements; 2) explicitly stated that the region was selected due to its documented history of severe and recurrent flooding; and 3) provided specific details on flood history and associated damages (e.g., major floods in 2015, 2019, 2022) and cited relevant sources (e.g., KWPA, 2019; Iranian Meteorological Organization, 2022), thereby clearly justifying its selection as a flood-prone case-study area.**

Materials and Methods – Model Description

6. Clarify how the parameters listed in Table 1 were selected. Provide relevant literature or scientific justification showing that these parameters influence flood susceptibility, supported by previous studies.

**Response: Table 1 is edited.**

7. The use of abbreviations is unclear. Ensure consistent and conscious use of abbreviations throughout the manuscript.

**Response: agree and change made for all abbreviations within the text.**

8. Explain how the parameters were obtained and inlcude the sources for each variable. Although the introduction notes that data were acquired from GEE, the methodology must clearly specify each parameter, its source, the time frame for any time-series data, data reliability (supported by previous applications of the datasets), spatial resolution, and any known limitations.

**Response: Regarding the variable Actual Evapotranspiration obtained from the dataset IDAHO_EPSCOR/TERRACLIMATE, the following information is provided:**

| **Name** | **Units** | **Min** | **Max** | **Scale** | **Pixel Size** | **Description** |
| --- | --- | --- | --- | --- | --- | --- |
| **aet** | **mm** | **0*** | **3140*** | **0.1 (Conversion Factor)** | **4000 Meters** | **Actual evapotranspiration, derived using a one-dimensional soil water balance model** |

- **Reference: Abatzoglou, J.T., S.Z. Dobrowski, S.A. Parks, K.C. Hegewisch, 2018, Terraclimate, a high-resolution global dataset of monthly climate and climatic water balance from 1958-2015, Scientific Data 5:170191, doi:10.1038/sdata.2017.191**

**The unit for the Aspect variable has been replaced with: ° (0–360).**

**Regarding the variable Soil Bulk Density obtained from the dataset OpenLandMap/SOL/SOL_BULKDENS-FINEEARTH_USDA-4A1H_M/v02, the following information is provided:**

| **Name** | **Units** | **Min** | **Max** | **Scale** | **Pixel Size** | **Description** |
| --- | --- | --- | --- | --- | --- | --- |
| **b0** | **kg/m³** | **5*** | **185*** | **10 (Conversion Factor)** | **250 Meters** | **Soil bulk density at 0 cm depth** |

- **Reference: Tomislav Hengl. (2018). Soil bulk density (fine earth) 10 x kg / m-cubic at 6 standard depths (0, 10, 30, 60, 100 and 200 cm) at 250 m resolution (Version v02) [Data set]. Zenodo. 10.5281/zenodo.1475970**

**Regarding the variable Soil Clay Content obtained from the dataset OpenLandMap/SOL/SOL_CLAY-WFRACTION_USDA-3A1A1A_M/v02, the following information is provided:**

| **Name** | **Units** | **Min** | **Max** | **Pixel Size** | **Description** |
| --- | --- | --- | --- | --- | --- |
| **b0** | **% (kg/kg)** | **2*** | **100*** | **250 Meters** | **Clay content at 0 cm depth** |

- **Reference: Tomislav Hengl. (2018). Clay content in % (kg / kg) at 6 standard depths (0, 10, 30, 60, 100 and 200 cm) at 250 m resolution (Version v02) [Data set]. 10.5281/zenodo.1476854**

**For the variable Climate Water Deficit obtained from the dataset IDAHO_EPSCOR/TERRACLIMATE, the following information is provided:**

| **Name** | **Units** | **Min** | **Max** | **Scale** | **Pixel Size** | **Description** |
| --- | --- | --- | --- | --- | --- | --- |
| **def** | **mm** | **0*** | **4548*** | **0.1 (Conversion Factor)** | **4000 Meters** | **Climate water deficit, derived using a one-dimensional soil water balance model** |

- **Reference: Abatzoglou, J.T., S.Z. Dobrowski, S.A. Parks, K.C. Hegewisch, 2018, Terraclimate, a high-resolution global dataset of monthly climate and climatic water balance from 1958-2015, Scientific Data 5:170191, doi:10.1038/sdata.2017.191**

**For calculating the Elevation variable from the dataset CGIAR/SRTM90_V4, the following information is provided:**

| **Name** | **Units** | **Min** | **Max** | **Pixel Size** | **Description** |
| --- | --- | --- | --- | --- | --- |
| **elevation** | **m** | **-444*** | **8806*** | **90 meters** | **Elevation** |

- **Reference: Jarvis, A., H.I. Reuter, A. Nelson, E. Guevara. 2008. Hole-filled SRTM for the globe Version 4, available from the CGIAR-CSI SRTM 90m Database:** [**https://srtm.csi.cgiar.org**](https://srtm.csi.cgiar.org/)**.**

**For calculating the Vegetation Index variable from the dataset MODIS/061/MOD13A1, the following information is provided:**

| **Name** | **Units** | **Min** | **Max** | **Scale** | **Pixel Size** | **Wavelength** | **Description** |
| --- | --- | --- | --- | --- | --- | --- | --- |
| **NDVI** |  | **-2000** | **10000** | **0.0001 (Conversion Factor)** | **500 Meters** | **None** | **Normalized Difference Vegetation Index** |

- **Reference: Please visit LP DAAC 'Citing Our Data' page for information on citing LP DAAC datasets.**

**For calculating the Land Cover variable from the dataset Iran Land Cover Map v1 13-class (2017) (pixel size: 13 meters), its specifications are as follows:**

**The table for differentiating land cover types for this product is as follows:**

**Dataset Provider**

[**K. N. Toosi University of Technology LiDAR Lab**](https://en.kntu.ac.ir/laser-scanners-laboratory/)

**The Iran-wide land cover map was generated by processing Sentinel imagery within the Google Earth Engine Cloud platform. For this purpose, over 2,500 Sentinel-1 and over 11,000 Sentinel-2 images were processed to produce a single mosaic dataset for the year 2017. Then, an object-based Random Forest classification method was trained by a large number of reference samples for 13 classes to generate the Iran-wide land cover map.**

| **classification** | **10 meters** | **Classification** |
| --- | --- | --- |

**classification Class Table**

| **Value** | **Description** |
| --- | --- |
| **1** | **Urban** |
| **2** | **Water** |
| **3** | **Wetland** |
| **4** | **Kalut (yardang)** |
| **5** | **Marshland** |
| **6** | **Salty Land** |
| **7** | **Clay** |
| **8** | **Forest** |
| **9** | **Outcrop** |
| **10** | **Uncovered Plain** |
| **11** | **Sand** |
| **12** | **Farm Land** |
| **13** | **Range Land** |

**For the variables Palmer Drought Severity Index, Potential Evapotranspiration, Cumulative Precipitation, Surface Soil Moisture, and Daily Minimum and Maximum Temperature, the dataset IDAHO_EPSCOR/TERRACLIMATE was used. The information about this dataset and the variables is as follows:**

| **Name** | **Units** | **Min** | **Max** | **Scale** | **Pixel Size** | **Description** |
| --- | --- | --- | --- | --- | --- | --- |
| **pdsi** |  | **-4317*** | **3418*** | **0.01** | **Meters**  **4000** | **Palmer Drought Severity Index** |
| **pet** | **mm** | **0*** | **4548*** | **0.1** | **Meters**  **4000** | **Reference evapotranspiration (ASCE Penman-Montieth)** |
| **pr** | **mm** | **0*** | **7245*** |  | **Meters**  **4000** | **Precipitation accumulation** |
| **soil** | **mm** | **0*** | **8882*** | **0.1** | **Meters**  **4000** | **Soil moisture, derived using a one-dimensional soil water balance model** |
| **tmmn** | **°C** | **-770*** | **387*** | **0.1** | **meters** | **Minimum temperature** |
| **tmmx** | **°C** | **-670*** | **576*** | **0.1** | **meters** | **Maximum temperature** |

- **Abatzoglou, J.T., S.Z. Dobrowski, S.A. Parks, K.C. Hegewisch, 2018, Terraclimate, a high-resolution global dataset of monthly climate and climatic water balance from 1958-2015, Scientific Data 5:170191,** [**doi:10.1038/sdata.2017.191**](https://doi.org/10.1038/sdata.2017.191)

**For the variable Topsoil Sand Percentage, the dataset OpenLandMap/SOL/SOL_SAND-WFRACTION_USDA-3A1A1A_M/v02 was used, and its specifications are as follows:**

| **Name** | **Units** | **Min** | **Max** | **Pixel Size** | **Description** |
| --- | --- | --- | --- | --- | --- |
| **b0** | **% (kg / kg)** | **1*** | **100*** | **Meters**  **250** | **Sand content at 0 cm depth** |

- **Tomislav Hengl. (2018). Sand content in % (kg / kg) at 6 standard depths (0, 10, 30, 60, 100 and 200 cm) at 250 m resolution (Version v02) [Data set].** [**10.5281/zenodo.1476851**](https://doi.org/10.5281/zenodo.1476851)

**For the Transpiration variable, the dataset NASA/FLDAS/NOAH01/C/GL/M/V001 was used, and its specifications are as follows:**

| **Name** | **Units** | **Pixel Size** | **Description** |
| --- | --- | --- | --- |
| **Evap_tavg** | **kg/m^2/s** | **Meters**  **11000** | **Evapotranspiration** |

- **McNally, A., Arsenault, K., Kumar, S., Shukla, S., Peterson, P., Wang, S., Funk, C., Peters-Lidard, C.D., & Verdin, J. P. (2017). A land data assimilation system for sub-Saharan Africa food and water security applications. Scientific Data, 4, 170012.**

**Regarding the Soil Texture variable, the dataset OpenLandMap/SOL/SOL_TEXTURE-CLASS_USDA-TT_M/v02 was used. The specifications of this product are as follows:**

| **Name** | **Pixel Size** | **Description** |
| --- | --- | --- |
| **b0** | **Meters**  **250** | **Soil texture class (USDA system) at 0 cm depth** |

**The table of specifications for the different soil texture classes based on this product is as follows:**

| **Value** | **Description** |
| --- | --- |
| **1** | **Cl** |
| **2** | **SiCl** |
| **3** | **SaCl** |
| **4** | **ClLo** |
| **5** | **SiClLo** |
| **6** | **SaClLo** |
| **7** | **Lo** |
| **8** | **SiLo** |
| **9** | **SaLo** |
| **10** | **Si** |
| **11** | **LoSa** |
| **12** | **Sa** |

**The slope variable was also calculated from the Digital Elevation Model (DEM) variable through coding in Google Earth Engine.**

**In response to the reviewers' comments, the following figures have been provided. Regarding the timing of the images, the figures below show the date of April 8, 2019, based on Sentinel-2 imagery.**

**Also, other auxiliary variables, as shown in the figure below (based on Sentinel-2 imagery), have been used to identify the target variable (flood-prone and non-flood-prone areas).**

**In addition, other auxiliary variables, such as those shown in the figure below (based on Sentinel-2 imagery), have been used to identify the target variable (flood-prone and non-flood-prone areas).**

**Furthermore, Figure 1 has been updated. In this figure, Sentinel-2 images have been added, and the NDWI index image for April 8, 2019, along with ground control points, has been included. The above content has been provided for the esteemed reviewers' reference.**

9. Figure 2 is comprehensive, but the accompanying narrative does not sufficiently describe the methodology. Section 2.2 would benefit from clearer methodological explanations, including workflow, step-by-step processes, input/output descriptions, software or tools used, and connections to the technical details provided later.

**Response: agree and change made**

10. Several abbreviations are not defined in the main text. Ensure that each abbreviation is defined at its first appearance (preferably in the main text rather than the abstract).

**Response: agree and change made for all abbreviations within the text.**

11. The models and methods used in the feature-selection stage (e.g., Boruta, Boruta-SHAP) are not introduced in the methodology. Include brief explanations of each method and clarify how they operate within your workflow.

**Response: Thank you for your valuable comment. We have added a new dedicated subsection, "2.3 Ensemble Feature Selection," to the Methodology. This section provides a concise, point-by-point introduction to each of the nine feature selection methods used (including Boruta, Boruta-SHAP, Elastic-Net, etc.), citing their foundational references. It clearly explains their operational principles and, most importantly, details how they were integrated into our workflow through a majority-vote consensus rule to determine the final robust feature set for the LSTM model. This addition ensures the feature selection stage is now transparently documented.**

12. The introduction to optimisation algorithms is good, but more technical detail is recommended. Include explanations of how each algorithm works (equations or schematic plots where appropriate) and how they were applied (e.g., for hyperparameter tuning).

**Response: In the final revision of Section 2.2, we added the core position-update equations (with citations) for all five metaheuristic algorithms (GWO, WOA, CSA, OOA, HOA). We also explicitly detailed their application for tuning specific LSTM hyperparameters (e.g., number of units, learning rate), defined the hyperparameter search space, and provided a structured table of each algorithm's control parameters and configuration. These revisions provide the requested technical detail and clearly link each algorithm's mechanism to its role in our optimization workflow.**

13. The rationale for using multiple feature-selection methods requires stronger justification. Begin by emphasising why multiple methods are necessary for identifying critical parameters, how each method differs conceptually, and how some can capture relationships better than others. Selecting parameters that consistently appear across methods is acceptable, but this requires clearer scientific reasoning and justification.

**Response: The use of multiple feature selection methods in this study represents a systematic, cautious, and scientific approach. In response to the question raised, the following addresses the necessity, advantages, conceptual differences, and scientific justification for employing several methods, with an emphasis on these points:**

**Regarding the rationale for using multiple methods, we respectfully submit the following to the esteemed reviewer:**

**Machine learning models are heavily influenced by the quality and structure of input data. Selecting irrelevant or highly correlated features not only reduces accuracy but also leads to overfitting, increases computation time, and diminishes model interpretability. However, no single method can definitively provide the most comprehensive perspective on the structure of data relationships because:**

- **Environmental data (such as PDSI, NDVI, TMMN) are inherently nonlinear, dynamic, and have complex correlations.**
- **Different methods operate based on distinct mathematical, statistical, and algorithmic principles. Each excels at uncovering specific types of relationships (linear, nonlinear, interactive, directional, or non-directional).**
- **A single method may, due to its inherent algorithmic bias, overlook important variables or erroneously select low-value variables.**

**Therefore, using a wide spectrum of methods—from statistical (e.g., Mutual Information) to model-based (e.g., Permutation Importance) to hybrid (e.g., Boruta-SHAP)—enables the following:**

- **Discovery of hidden and nonlinear relationships.**
- **Evaluation of the stability of selected features.**
- **Reduction of the risk of decision-making based on a single feature selection model. (Can we definitively say which feature selection model's results are the best and, consequently, that the optimal dataset for machine learning was chosen? This would lead to high uncertainty and constitute a significant risk).**

**As the esteemed reviewer pointed out, there are key conceptual differences between the methods used. Some feature selection models, by their very nature, tend to select specific types of data and variables as optimal, and bias in feature selection can exist due to the models' intrinsic properties. The foundations and nature of several of these feature selection models are outlined below:**

| **Method** | **Evaluation Basis** | **Discoverable Relationship Type** | **Key Strengths** | **Potential Limitations** |
| --- | --- | --- | --- | --- |
| **Boruta** | **Comparison with Shadow Features using Random Forest** | **Nonlinear, Interactive** | **Resistant to noise; identifies truly relevant features** | **May have lower sensitivity to features with weak combined effects** |
| **Boruta-SHAP** | **Combines Boruta with SHAP (Shapley Additive Explanations) values** | **Nonlinear + Local/Global interpretability** | **More precise importance ranking; greater transparency in feature impact** | **Computationally intensive; dependent on base model (usually tree-based)** |
| **Elastic-Net** | **Combination of L1 (Lasso) and L2 (Ridge) Regularization** | **Primarily linear (with limited capacity for nonlinear)** | **Prevents overfitting; automatic selection + compression** | **May underestimate important nonlinear features (e.g., NDVI in certain ranges)** |
| **Mutual Information (MI)** | **Measures nonlinear information shared between feature and target** | **Nonlinear, non-directional** | **Model-agnostic; sensitive to complex relationships** | **Cannot directly model multivariate interactions** |
| **Permutation Importance** | **Reduction in model performance after randomly shuffling each feature** | **Any relationship the model can learn** | **Dependent on the final model; interpretable from a performance perspective** | **Dependent on the base model; can be misleading in the presence of strong feature dependencies** |
| **RFE** | **Recursive elimination based on model weights (e.g., SVM or RF)** | **Dependent on base model** | **Efficient for dimensionality reduction; suitable for linear/tree models** | **Can exhibit unstable behavior in the presence of multicollinearity** |
| **SFS** | **Incremental addition based on R² or performance metric** | **Optimal combinatorial search** | **Suitable for finding the smallest optimal subset** | **Computationally very expensive; prone to getting stuck in local optima** |
| **Stability Selection** | **Repeated selection on different data subsets + probability threshold** | **Model-agnostic (usually with Lasso or RF)** | **Highly resistant to data fluctuations; reduces Type I error** | **May discard stable but low-impact variables** |
| **Deep Learning (ANN)** | **Input gradients or Sensitivity Analysis** | **Highly complex nonlinear** | **Learns high-level interactions automatically** | **Black box; requires large data; sensitive to architecture and training** |

**Regarding the rationale for using Intersection as the final strategy, the following justification is respectfully submitted:**

**Using the intersection of features selected by all/most methods is a consensus-based strategy widely accepted in fields like environmental modeling. The scientific reasons are:**

**A) Reduction of False Positives (Type I Error)
Each method may, due to its inherent bias, falsely identify an unimportant variable as significant. However, the probability that all or most independent methods would coincidentally select the same spurious variable is very low. For example, consider these variables:**

- **NDVI and TMMN: Selected by 9/9 methods → a very high probability of a real association with the target variable.**
- **Pr and moisture: Selected by 7/9 methods → strong evidence for a real association.**
- **Landscape: Selected by 0/9 methods → sufficient evidence to reject a strong association with the target. Relying on a single method might have incorrectly included this variable in the modeling dataset.**

**B) Increased Generalizability
Features that are consistently present across model-based (Permutation, DL), statistical (MI), and hybrid (Boruta-SHAP) methods are likely to:**

- **Not be intrinsically dependent on a specific algorithm.**
- **Be stable under different data conditions (linear/nonlinear, sparse/dense).**
- **Be physically/environmentally interpretable (e.g., NDVI: vegetation health related to flooding).**

**C) Alignment with Domain Knowledge
The consensus result—prioritizing NDVI, TMMN, Pr, and moisture—is fully consistent with current flood literature.**

**In conclusion, why is this approach superior?
As known, diversity of methods creates diversity of perspectives. Consensus among diverse perspectives is the best approximation of reality in situations where:**

- **The phenomenon (flood zoning) is multivariate and nonlinear.**
- **The data have complex correlations and sometimes contain noise.**
- **The goal is to create an interpretable, stable, and generalizable model.**

**Therefore, relying on a single method, even an advanced one, carries significant scientific risk. In contrast, the multi-method approach with consensus inference:**

- **Increases the reliability of the results.**
- **Enhances methodological transparency.**
- **Provides a solid foundation for subsequent decision-making (e.g., final modeling or policymaking).**

**Thus, using multiple different methods is not merely an innovation or redundancy, but a methodological necessity for achieving robust, well-founded, and scientifically defensible results.**

14. The methodology does not explain how flood susceptibility was calculated or defined. Clarify whether susceptibility is based on simulated flood extents, observed flood events, or other criteria. Given that this is the model’s target variable, a detailed explanation is essential.

**Response: In the present study, the target variable was not indices of flood vulnerability, etc. Rather, it was the visual observation of flood events over several days on satellite imagery and the classification of these zones into flooded and non-flooded areas, which were then used as target variables in the modeling process.**

15. The manuscript does not clearly explain how LSTM is utilised in the model. Specify whether the model was trained using sequential time-series data and how changes in each parameter relate to flood susceptibility over time. This should be clarified before discussing optimisation.

**Response: In the present study, from April 13, 2019, and for several subsequent days during which water remained stagnant in the study area and rainfall continued, LSTM models were used based on multiple images (the target variable was a sequential time series for the periods of stagnant water presence and observed flood zones on satellite imagery). All independent variables were also averaged over the study's time period. Finally, to display flood-prone and non-flood-prone zones, the results were averaged.**

**In response to the reviewers' comments, the following figures have been provided. Regarding the timing of the images, the figures below show the date of April 8, 2019, based on Sentinel-2 imagery.**

**Also, other auxiliary variables, as shown in the figure below (based on Sentinel-2 imagery), have been used to identify the target variable (flood-prone and non-flood-prone areas).**

**In addition, other auxiliary variables, such as those shown in the figure below (based on Sentinel-2 imagery), have been used to identify the target variable (flood-prone and non-flood-prone areas).**

**Furthermore, Figure 1 has been updated. In this figure, Sentinel-2 images have been added, and the NDWI index image for April 8, 2019, along with ground control points, has been included. The above content has been provided for the esteemed reviewers' reference.**

16. The target variable requires a clearer definition. Indicate whether it represents flooded/not-flooded classes, flood context, susceptibility scores, or another metric.

**Response: The target variables are flood-prone and non-flood-prone (or flooded and non-flooded) zones (coded as 1 and 0) at the time of the flood event (based on satellite imagery). No vulnerability index was used.**

Results

17. There is no need to provide multiple figures and tables for each feature-selection model, as most of them yield similar conclusions. Include only the most relevant figures and provide a consolidated summary of results.

**Response: Given that the study utilized the collective results of the feature selection models (their intersections in selecting variables) to ultimately identify the best variables for input into machine learning, the results of all models were reported. In this research, instead of using a single feature selection method, multiple models were employed—a form of innovation and comprehensive analysis. Each model yielded different results in terms of both variable prioritization and variable type. Therefore, the optimal approach was to select the variables common across the different feature selection methods in order to achieve the best estimation regarding the target variable (flood-prone and non-flood-prone zones).**

Overall Comments

18. The manuscript lacks several essential details. Consider explicitly defining flood susceptibility and the approach used to calculate or evaluate it; explaining how LSTM was applied, including the structure of sequential data; and clearly defining the target variable.

**Response: In the present article, based on Sentinel 2 images from the dates of the flood event (Sentinel 2 OLI sensor, dated April 13, 2019, and several days following the event, considering water stagnation), flooded and non-flooded (flood-prone and non-flood-prone) areas were delineated using visual observation of the aforementioned images. This layer served as the target variable and was incorporated into the modeling process with codes 0 and 1.**

**Furthermore, all other variables for the period of the flood event were averaged using Google Earth Engine (GEE) data, converting them into single images (e.g., precipitation, evapotranspiration, etc.) or were inherently single images (e.g., slope, aspect, etc.). In other words, the dynamic and non-dynamic nature of the data was considered for temporal harmonization.**

**Subsequently, based on the pixel size of the Landsat images and the extent of the study area, sampling points were generated (all cells of the Landsat image, cropped to the study area, were converted to points). Sampling was performed for all the mentioned variables (the images had been resampled prior to sampling).**

**The collected samples, which included both the target variable values and those of other variables in their database, were then fed into the feature selection processes. Through the examination of various methods, the best variables were identified as predictors for the target variable to proceed to the machine learning modeling stages. At this stage, the consensus (intersection) of multiple feature selection models was considered, rather than relying solely on the results of a single model (the collective results of all models were taken into account).**

**In subsequent stages, both internal model evaluations based on performance metrics and evaluations of the models against ground truth (observations of flood-prone and non-flood-prone areas) were conducted.**

19. The manuscript would benefit from substantial language improvement. Abbreviations are inconsistently defined, and several incorrect terms (e.g., “Natural Disasters”) remain. Some grammatical errors are also present. Reviewers strongly discourage relying solely on generative AI for language refinement.

**Response: agree and change made for all abbreviations within the text.**
