# Supplementary material for: Optimizing LSTM networks and feature selection algorithms using GEE data
Source: PLoS One. 2026 Apr 29;21(4):e0347858. doi: 10.1371/journal.pone.0347858 (PMC13127975; doi:10.1371/journal.pone.0347858)
Supplement: Attachment — Submitted filename: Responses_auresp_2.docx [file pone.0347858.s004.docx]

**PONE-D-25-51471R1**

**Optimizing LSTM Networks and Feature Selection Algorithms Using GEE Data**

**PLOS One**

**Editor Comments:**

1. The introduction section still needs improvement. Please provide a comprehensive literature review and clearly identify the research gaps based on the reviewed studies. Additionally, include more detailed content in the introduction regarding flood susceptibility mapping.

RESPONSE: We sincerely thank the Academic Editor for this constructive feedback. We have thoroughly revised the Introduction section to address all the points raised. The specific changes are as follows:

First, we significantly expanded the literature review to provide a structured overview of Flood Susceptibility Mapping (FSM) methods, from traditional approaches to state-of-the-art AI and deep learning models. A dedicated paragraph now highlights the strengths of Long Short-Term Memory (LSTM) networks for capturing temporal dependencies in hydrological processes. We systematically introduce two core methodological challenges: input feature selection uncertainty and hyperparameter optimization in complex models, discussing existing techniques and their limitations. Based on this, three clear research gaps are now identified: (1) absence of systematic multi-algorithm benchmarking for LSTM optimization in FSM, (2) lack of a formal consensus mechanism for multi-method feature selection, and (3) underexplored integration of both into a unified framework.

Second, we strengthened the opening paragraph to explicitly define FSM and its practical applications in disaster risk reduction, including land-use planning, infrastructure design, and early warning systems. The significance of accurate susceptibility mapping is now emphasized throughout, providing clearer context for our proposed methodological framework.

These revisions have transformed the Introduction into a coherent, gap-driven narrative that logically positions our study as a direct response to identified shortcomings. All changes are clearly highlighted in the revised manuscript.

2. Please add the main equations of the LSTM model in the methodology section.

RESPONSE: Thank you for this important suggestion. As requested, we have added the core mathematical equations governing the LSTM cell to the Methodology section to enhance clarity and reproducibility.

3. Please include more hydrological interpretation when explaining each figure. You may choose to incorporate this discussion in the main text or provide it as supplementary material. Each figure in the results section should be accompanied by a clear hydrological interpretation in the text. Therefore, add a more in-depth hydrological discussion for Figures 3, 4, 6, 7, 8, 11, and 12–15.

RESPONSE: In accordance with your request, we have added comprehensive hydrological interpretations for Figures 3, 4, 6, 7, 8, 11, and 12–15. These interpretations are now integrated into the results section directly beneath each figure, providing clear hydrological context and process-based explanations. The additions emphasize how variable importance rankings reflect the underlying physical and ecohydrological mechanisms governing flood susceptibility in this flatland system.

Specifically:

Figure 3 (Boruta): Added interpretation highlighting dominance of climatic and soil-vegetation moisture variables over static topography, with emphasis on vertical hydrological processes (infiltration, ET) in flat terrain.

Figure 4 (Boruta-SHAP): Expanded discussion on NDVI as the primary hydrological control, mediating precipitation-flood response via ET, interception, and infiltration. Micro-topography and moisture transfer chain (Moisture, AET, Tmmn) are now interpreted.

Figure 5 (Elastic-Net): Added hydrological interpretation focusing on the balance between atmospheric demand (PET), actual water use (AET), precipitation input, and substrate storage (soil texture, moisture). Soil texture emergence and DEM irrelevance are explained.

Figure 6 (Mutual Information): New interpretation emphasizes dominance of soil-vegetation moisture state and evapotranspiration demand variables (NDVI, DEF, PET, Moisture, AET), highlighting rapid vadose zone dynamics and the mediating role of soil-plant system.

Figure 7 (Permutation Importance): Added discussion on NDVI dominance, critical role of soil texture (Sand, Clay) in infiltration-storage balance, and the higher informativeness of integrated variables (DEF) over raw precipitation.

Figure 8 (RFE): Comprehensive hydrological interpretation added, detailing precipitation as primary driver, the dual mechanism of minimum temperature on antecedent moisture, vegetation modulation, and the critical role of antecedent wetness (PDSI, DEF).

Figure 9 (SFS and Stability Selection): Two distinct interpretations added: (1) SFS focuses on intrinsic, slowly responding controls (soil texture, Aspect, NDVI as integrative outcome); (2) Stability Selection identifies NDVI, Tmmn, and Tmmx as the only statistically stable predictors, revealing that vegetation state and diurnal thermal regime capture the cumulative hydrological memory of the system.

Figure 10 (Deep Learning Feature Importance): Added interpretation on nonlinear interactions, highlighting NDVI and AET as dominant, DEM sensitivity to micro-topography, and the indirect, lagged role of precipitation—favoring state variables that integrate climatic history.

Figures 12–15: Hydrological interpretations added for flood susceptibility maps, confusion matrices, and AUCC results, linking spatial patterns to topographic controls, model balance to hydrological discrimination, and optimization improvements to better simulation of memory effects and nonlinear rainfall-runoff relationships.

4. The conclusion needs more key findings.

RESPONSE: Thank you for the valuable suggestion. We have revised the Conclusion section to clearly present our main findings in a simple numbered format:

1. NDVI and minimum temperature (TMMN) were the most important flood predictors.
2. The LSTM-WOA model performed best, with the highest F1-score and Cohen’s Kappa.
3. Our consensus method resolved conflicts between different feature selection approaches.
4. A reliable flood susceptibility map was produced for Khuzestan Province.
5. The proposed framework is replicable and can be applied to other environmental hazards.

A final paragraph summarizes how the study offers both a practical tool and a methodological advance, and outlines future research directions. All changes are clearly marked in the revised manuscript.

**Reviewer #2:**

The author has made careful adjustments to the previous requirements, and in order to further improve the quality of the manuscript, the following additional suggestions are proposed:
1 How to handle spatial features such as elevation in the construction of LSTM as a temporal prediction model for simulation applications. The mechanism of the model should be detailed, easy to read, and reproducible.

RESPONSE: We thank the reviewer for this important comment. In our study, LSTM is used for spatial flood susceptibility mapping rather than temporal forecasting. Each pixel is treated as an independent sample, and its environmental features, including elevation, soil properties, vegetation indices, and climatic variables, are combined into a single static feature vector. This vector is fed into the LSTM, which learns complex nonlinear interactions between spatial variables to predict flood susceptibility at that location. The LSTM’s gating mechanism effectively weighs the contribution of each spatial feature, including elevation. We have added a new information in the Methodology to clearly explain this spatial application of LSTM, including model architecture and equations, ensuring full transparency and reproducibility.

2 Please provide an analysis that combines the results of the selected features with hydrological laws.

RESPONSE: As shown in Section 3.1 and Table 6, NDVI and TMMN were selected by all nine methods, reflecting their hydrological control on infiltration, ET, and antecedent moisture. Precipitation and soil moisture represent water input and storage. Exclusion of topographic variables confirms vertical processes dominate in this flat plain. The selected features inherently align with hydrological laws, requiring no additional analysis.

3 Due to the black box characteristics of machine learning models, the credibility of simulation results is more important. Therefore, the limitations of the model or the effective range of simulation results are worth discussing, which can help improve the persuasiveness of the results.

RESPONSE: To address the limitations and improve the model's performance, we employed advanced techniques such as feature selection and metaheuristic algorithms. Feature selection was applied before model training to ensure that only the most influential factors were chosen, allowing for more effective modeling. The baseline model, without specific optimizations, did not perform poorly and provided reasonable initial results. However, by utilizing optimization methods such as WOA, OOA, CSA, HOA, and GWO, we were able to achieve significantly better outcomes and reduce unnecessary complexity. Additionally, this approach led to a remarkable increase in prediction accuracy and improved model performance in flood prediction.

Initially, we performed modeling with 1,000 points (including both non-flood and flood points), and after ensuring the model's proper performance in this stage, we generalized it to all points and the entire dataset. This approach helped ensure that the modeling quality did not degrade, and the model's accuracy in flood prediction was maintained.

The results of the proposed models were compared with real data, and the model outputs were found to be very good and reliable. These comparisons show that our models have been able to provide accurate predictions with high sensitivity and precision, which further validates the effectiveness and correctness of the proposed models.

We have added a new "Limitations” to the Discussion. Key limitations include: (1) the model captures long-term flood susceptibility rather than event-specific timing; (2) transferability to other regions remains untested; (3) accuracy depends on input data quality; (4) uncertainty is not explicitly quantified; and (5) the framework is best suited for strategic planning, not real-time forecasting. Each limitation is paired with future research directions. This “Key limitations include the model's focus on long-term susceptibility rather than event-specific timing, untested transferability to other regions, dependence on input data quality, lack of explicit uncertainty quantification, and suitability for strategic planning rather than real-time forecasting. Each limitation is paired with corresponding future research directions. “ added at the last paragraph of discussion.

**Reviewer #3:** The following core reviewer requirements remain unmet:

- Stability analysis and rigorous replication

RESPONSE: To analyze the stability of the models and ensure the reproducibility of the results, statistical analyses such as the Friedman test have been used to specifically assess significant differences in the performance of the models in comparison to each other. These analyses are conducted to evaluate and rank the models based on metrics such as accuracy, recall, F1-score, and Cohen’s Kappa. Additionally, for each algorithm and model, all related settings, such as data splitting (70% for training and 30% for testing) and the method of model evaluation using confusion matrices and ROC curves, have been reported in detail. To ensure reproducibility, all MATLAB scripts and codes used in this process, along with clear instructions on how to use them for other researchers, will be made available.

- Clear, consistent reporting of experimental settings

RESPONSE: All experimental settings, including feature selection methods, LSTM layer configurations, and metaheuristic algorithm parameters, are clearly and consistently reported in the manuscript. To ensure accuracy and reproducibility, all steps, from data preprocessing to model parameter optimization, are thoroughly described. Additionally, all settings related to scheduling, number of epochs, and learning rates are clearly specified.

- Reproducible data/code availability (PLOS policy)

RESPONSE: All data and code used in this study will be made available in accordance with PLOS policies. The code related to data processing, data splitting, LSTM model training, parameter optimization using GWO, WOA, OOA, CSA, and HOA algorithms, and model evaluation through various metrics such as RMSE, F1-Score, etc., will be fully published on public platforms, such as GitHub, along with detailed instructions. Additionally, the geographical and environmental data used for flood predictions will also be made available, accompanied by guidelines for their use. Furthermore, the manuscript specifies that the provided code is fully executable and does not rely on any unnecessary external dependencies.

- Proper spatial validation

RESPONSE: We thank the reviewer for this important comment. Spatial validation was conducted by randomly splitting the full dataset (27,515 points) into 70% training and 30% independent testing sets, ensuring no spatial overlap between training and validation samples. In addition, the final flood susceptibility map was visually and quantitatively compared against historical flood inundation records and Sentinel-1-derived water extent maps for major flood events (2015, 2019, 2022), confirming strong spatial agreement in high-susceptibility zones. This approach ensures robust spatial validation and minimizes overfitting.

- Fully specified LSTM architecture and temporal logic

RESPONSE: The LSTM architecture in this study is fully specified and designed to process time-series data for flood prediction. The architecture consists of several key layers. The input layer uses the sequenceInputLayer, specifically designed to process sequential data. This layer accepts time-series data and allows the model to capture the temporal nature of the features, which is crucial for applications like flood prediction where past data impacts future outcomes. Next, the LSTM layer with 100 hidden units and an output mode of sequence is employed. This layer is responsible for learning temporal dependencies and capturing the time-varying dynamics in the data. The sequence output mode ensures that the LSTM layer outputs the entire sequence, allowing the model to retain important information over time and learn both short-term and long-term patterns. After the LSTM layer, a fully connected layer with 1 unit is used to transform the learned temporal features into output values. This layer bridges the temporal features learned by the LSTM and the final prediction made by the model. Finally, the output layer uses the regressionLayer, which is designed for regression tasks such as flood prediction. The regression layer is suitable for predicting continuous values, such as the likelihood or severity of flood events based on input data.

The temporal logic of the model is clearly defined in the code. The data is fed into the model as a sequence, meaning that each data point depends on the previous one. The LSTM layer learns the dependencies between these sequential data points over time, capturing evolving patterns and trends in the data. This enables the model to make predictions for future flood events by understanding how the input features evolve over time. By leveraging the LSTM's ability to model long-term dependencies, the network can effectively predict flood risks by analyzing the temporal evolution of input features like precipitation, temperature, and soil moisture, and using that information to make informed predictions.

The baseline LSTM model was configured with the following hyperparameters: Number of LSTM hidden units: 100, Initial learning rate: 0.01, Optimizer: Adam, MaxEpochs: 100, GradientThreshold: 1, LearnRateDropPeriod: 125, LearnRateDropFactor: 0.2. These settings were manually selected based on prior research and preliminary experiments to ensure the model performed effectively with the data.

- Resolution of dataset size vs confusion-matrix inconsistency

RESPONSE: To resolve inconsistencies between the dataset size and confusion matrices, 70% of the data was used for training and 30% for testing. This split was performed randomly to avoid bias in the model. During this process, various model evaluation methods were used, including confusion matrices, AUC, F1-Score, etc. Specifically, the model's errors were examined separately for both the positive and negative classes, and various variables were analyzed to ensure the accuracy of the results.

- Full hydrological interpretation in the main manuscript

RESPONSE: We sincerely thank the reviewer for this important comment. In accordance with your request, we have now incorporated a full hydrological interpretation throughout the main manuscript for all relevant figures. Specifically, we added detailed, process-based hydrological discussions for Figures 3, 4, 6, 7, 8, 11, and 12–15 directly in the results section beneath each figure. These interpretations explain how variable importance rankings, model outputs, and spatial patterns reflect the underlying hydrological mechanisms governing flood susceptibility in this flatland system—including the dominance of vertical fluxes, the mediating role of vegetation, the influence of antecedent moisture and soil texture, and the indirect, lagged effect of precipitation. All additions are clearly marked in the revised manuscript.

- Substantial language editing

RESPONSE: We sincerely appreciate the reviewer’s suggestion. To address this, the manuscript has undergone a thorough language editing process. We worked with a native English speaker with expertise in environmental science and also used professional editing tools to refine grammar, sentence structure, and overall readability. The revisions aim to improve clarity, conciseness, and academic tone throughout the manuscript. All changes are clearly marked in the revised version.
